# Evaluation and Validation of Reference Genes for Quantitative Real-Time PCR in *Helopeltis theivora* Waterhouse (Hemiptera: Miridae)

**DOI:** 10.1038/s41598-019-49479-1

**Published:** 2019-09-16

**Authors:** Zheng Wang, Qianqian Meng, Xi Zhu, Shiwei Sun, Shengfeng Gao, Yafeng Gou, Aiqin Liu

**Affiliations:** 1 Hainan Provincial Key Laboratory of Genetic Improvement and Quality Regulation for Tropical Spice and Beverage Crops, Spice and Beverage Research Institute, Chinese Academy of Tropical Agricultural Sciences, Wanning, 571533 China; 2 Institute of Tropical Agriculture and Forestry, Hainan University, Haikou, 570228 China

**Keywords:** Transcription, Physiology

## Abstract

*Helopeltis theivora* Waterhouse is a predominant sucking pest in many tropic economic crops, such as tea, cocoa and coffee. Quantitative real-time PCR (qRT-PCR) is one of the most powerful tools to analyze the gene expression level and investigate the mechanism of insect physiology at transcriptional level. Gene expression studies utilizing qRT-PCR have been applied to numerous insects so far. However, no universal reference genes could be used for *H*. *theivora*. To obtain accurate and reliable normalized data in *H*. *theivora*, twelve candidate reference genes were examined under different tissues, developmental stages and sexes by using geNorm, NormFinder, BestKeeper, Delta Ct and RefFinder algorithms, respectively. The results revealed that the ideal reference genes differed across the treatments, and the consensus rankings generated from stability values provided by these programs suggested a combination of two genes for normalization. To be specific, *RPS3A* and *Actin* were the best suitable reference genes for tissues, *RPL13A* and *GAPDH* were suitable for developmental stages, *EF1α* and *RPL13A* were suitable for sexes, and *RPL13A* and *RPS3A* were suitable for all samples. This study represents the first systematic analysis of reference genes for qRT-PCR experiments in *H*. *theivora*, and the results can provide a credible normalization for qRT-PCR data, facilitating transcript profiling studies of functional genes in this insect.

## Introduction

Quantitative real-time polymerase chain reaction (qRT-PCR) was developed and became been widely used in modern biology and biomedical sciences, due to the technical advantages of high sensitivity and repeatability, more rapidity and possibility to investigate several target genes simultaneously^1,2^. This method has been applied in many research directions, including amplification specificity and mRNA expression level detection, restriction fragment length polymorphism (RFLP) and single nucleotide polymorphism (SNP) analysis, etc^3,4^. However, the accuracy of quantitative analysis can be influenced by the quality and completeness of RNA and cDNA and amplification and experimental procedures among treatments^5,6^. Therefore, it is essential to minimize the biases by qRT-PCR normalization using internal reference genes^7–9^. Generally, internal reference genes are derived from housekeeping genes, which roles are maintaining the basic processes of cell cycle. So they are a class of genes expressed relatively stable in theory^3,10,11^. Nonetheless, the housekeeping genes expression levels is variable substantially in different treatments^12,13^. Hence, a particular and integrated research for each targeted species and experimental background is recommended for the validation of appropriate reference genes^14,15^.

The selection of a subset of reference genes that are constitutively expressed in various experiment conditions, including biotic and abiotic factors, is the first committed step for the accurately quantitative analysis of gene expression by qRT-PCR^2,16^. So plenty of publicly available online statistical tools, such as geNorm^7^, NormFinder^17^ and BestKeeper^18^, have been developed to identify a series of applicable housekeeping genes as internal controls for qRT-PCR normalization. At present, several reference genes have been identified under certain circumstance in Hemipteran insects, including ribosomal protein S9 (*RPS9*), elongation factor 1 alpha (*EF1α*) and TATA-box binding protein (*TBP*) for *Aphis glycines*^19^, *EF1α*, ribosomal protein L5 (*RPL5*) and *RPS18* for *Bactericera cockerelli*^20^, *β-tubulin1*, *β-tubulin2* and succinate dehydrogenase subunit A1 (*SdhA1*) for *Ericerus pela*^21^, 16S ribosomal RNA(*16SRNA*), *RPS18* and *RPL13* for *Lipaphis erysimi* (Kaltenbach)^22^, *β-tubulin* and *EF1α* for *Bemisia tabaci* (Gennadius)^23^.

*Helopeltis theivora* Waterhouse (Hemiptera: Miridae) is an important pest of many economic crops, including tea (*Camellia sinensis* L.), cashew (*Anacardium occidentale* L.), cocoa (*Theobroma cocoa* L.), coffee (*Coffea* spp.), pepper (*Piper nigrum* L.), cinchona (*Cinchona* spp.) and guava (*Psidium guajava* L.), to which it causes considerable damages^24^. Both adults and nymphs of *H*. *theivora* take in the plant juice from young buds, leaves, fruits and shoots with their piercing-sucking mouthparts^25^. As a consequence, drying up of shoots and fruits can arise from their sucking behavior, which causes more than 25% of crop losses^26,27^. In addition, this pest demonstrates high persistence of populations against stressful environment through strong ability of adaptation. However, the molecular biology of *H*. *theivora*, including the functional study of target genes and physiological mechanisms of its adaptability, is less clarified so far. Lack of suitable reference gene may be a major hurdle for the deeper studies in this insect. So the objective of this study was to assess and validate the most applicable reference genes for data normalization of qRT-PCR under various conditions in *H*. *theivora*. Twelve candidate genes from five functional classes were identified and cloned, including 3 structure-related genes (*Actin*, *β-Tubulin1* and *β-Tubulin2*), 3 ribosomal genes (*RPL13A*, *RPS3A* and *18SRNA*), 3 protein production genes (*EF1α*, eukaryotic initiation factor 4A (*EIF4A*) and ubiquitin-conjugating protein (*UBQ*)), 2 metabolism-related genes (glyceraldehyde-3-phosphate dehydrogenase (*GAPDH*) and glucose 6 phosphate dehydrogenase (*G6PDH*)), and 1 transcription factor gene (*TBP*). The expression stability of these genes in sixteen samples (six from tissues, six from developmental stages and four from sexes) was performed by five Microsoft Excel-based programs (geNorm, NormFinder, BestKeeper, Delta Ct and RefFinder). The outcome of the study will not only benefit the future quantification of gene expression in *H*. *theivora* but will find translational application in other closely related Mirid.

## Results

### Primer evaluation and amplification efficiency of candidate reference genes

The specificity of all the primers was validated by single amplification band of expected size and melt curve in RT-PCR and qRT-PCR, respectively (Figs S1 and S2). The primer amplification efficiency and linear correlation coefficient (*R*^2^), which met the standard requirement of conventional qRT-PCR, were determined by the standard curve. The primer efficiency ranged from 90.1 to 96.9% and the *R*^2^ was above 0.998 (Table 1).

**Table 1:** Primer amplification characteristics of candidate genes for qRT-PCR in *H*. *theivora*.

Gene (Abbreviation)	Accession Number	Primer sequence	Product length (bp)	Tm (°C)	*R* ^2^	E (%)
*Actin*	MN027587	5′ TACCGCAAGATTCCATACCCA 3'	109	60	0.999	90.58
		5′ AAGTCCTACGAGCTTCCCGAC 3'				
*β-Tubulin1*	MN027588	5′ GATGAAGGAAGTTGACGAGCA 3'	237	57	0.999	90.10
		5′ TCACCAGTATACCAATGGAGG 3'				
*β-Tubulin2*	MN027589	5′ TCAGGAAAAGGAGGCGGCAAA 3'	365	61	0.999	90.99
		5′ GGCGAGGGAACGACAGAGTAG 3'				
*RPL13A*	MN027592	5′ ACACAAAATCGAAAGGGGAAA 3'	153	59	1.000	93.76
		5′ CGACCAAGATGACAGTAGGCA 3'				
*EF1α*	MN027594	5′ TCGCTTTCGTTCCAATCTCTG 3'	107	60	0.999	91.66
		5′ GGCTTTGCCTTCTTTCCTTTC 3'				
*RPS3A*	MN027598	5′ GCAGCATGGTGAAAAAGTGGC 3'	151	60	0.999	91.21
		5′ ACCTGGTTGTGTTGAGCGTAA 3'				
*GAPDH*	MN027590	5′ CAATGACCCCTTTTTGGGCG 3'	163	60	0.999	96.69
		5′ CCATGGGATGGCTTTGGGAT 3'				
*18SRNA*	MN027591	5′ GCGATGTTGAACGTTGCTGT 3'	110	59	0.999	96.32
		5′ ACGCTTATGGCTCCGTCAAA 3'				
*G6PDH*	MN027593	5′ CGTCTGCATATAGCGTCCGT 3'	237	60	0.999	95.01
		5′ GAGAACCACGTCGTCCACAT 3'				
*EIF4A*	MN027595	5′ TGGTTCGAAACATGCCGAGA 3'	184	60	0.999	93.64
		5′ GCGTTGCTGAATAGCGGAAG 3'				
*TBP*	MN027596	5′ CATCCCCAGCATCGGTACTC 3'	296	60	0.999	93.79
		5′ AAGGCGTCATCGGTGTCATT 3'				
*UBQ*	MN027597	5′ GATTATCCGCTGAGCCCTCC 3'	226	60	1.000	96.87
		5′ CATTAGCGCCACTTTCGTCG 3'				

### Expression profiles of candidate reference genes

**Figure 1:**
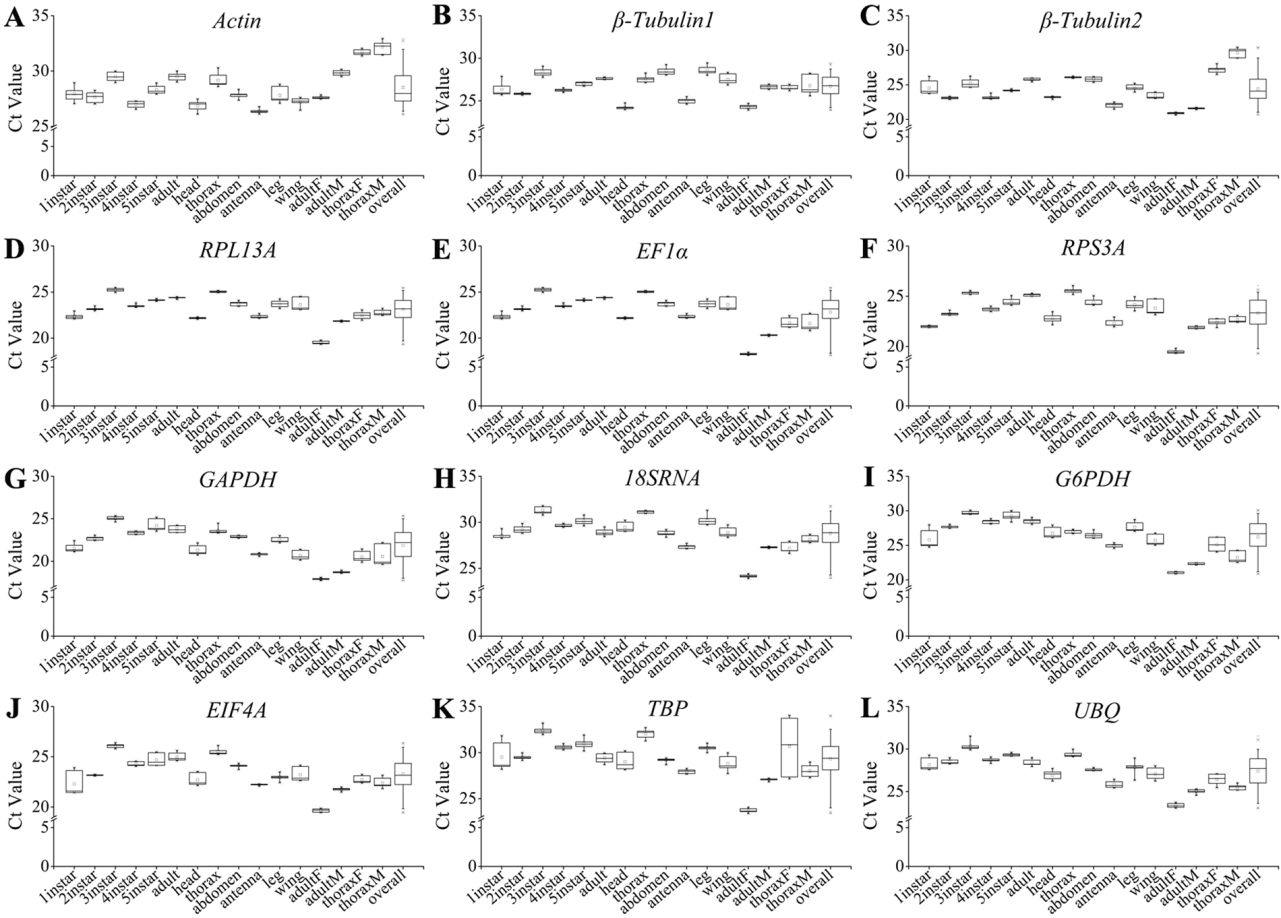
Average Ct values of twelve candidate reference genes at different tissues, development stages and sexes in *H*. *theivora*.

Expression levels of all candidate reference genes in sixteen samples (six tissues, six development stages and four sexes) were detected by qRT-PCR and summarized as Box-Whisker plots. The raw Ct values are available in Table S2. In all samples, *GAPDH* was found to be the most abundant with the lowest mean Ct value at 21.89 ± 1.98, whereas *TBP* transcript showed the lowest level with the highest mean Ct value at 29.33 ± 2.08 (Fig. 1G,K). Other candidate genes were also expressed at moderate levels, with mean Ct values at 28.52 ± 1.67, 26.69 ± 1.36, 24.41 ± 2.22, 23.13 ± 1.40, 22.87 ± 2.00, 23.33 ± 1.56, 28.78 ± 1.75, 26.23 ± 2.46, 23.30 ± 1.60, and 27.40 ± 1.83 in response to *Actin*, *β-Tubulin1*, *β-Tubulin2*, *RPL13A*, *EF1α*, *RPS3A*, *18SRNA*, *G6PDH*, *EIF4A* and *UBQ* (Fig. 1). The largest and smallest dispersion of Ct values were identified for *β-Tubulin1* gene at 4.44 cycles and *Actin* gene at 2.82 cycles in tissues (Fig. 1A,B), *G6PDH* gene at 3.88 cycles and *UBQ* gene at 2.23 cycles in developmental stages (Fig. 1I,L), *β-Tubulin2* gene at 8.75 cycles and *β-Tubulin1* gene at 2.47 cycles in sexes, and *β-Tubulin2* gene at 8.75 cycles and *β-Tubulin1* gene at 4.44 cycles in all samples, respectively (Fig. 1B,C).

### Selection of credible reference genes

#### geNorm analysis

**Figure 2:**
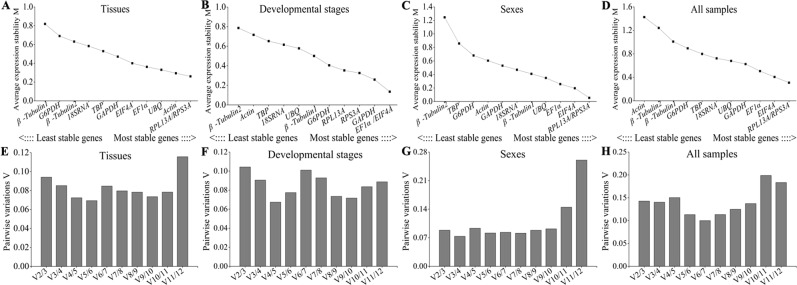
Expression stability and optimal number for normalization of candidate reference genes analyzed by geNorm. **(A**–**D)** The stability M values of candidate genes in tissues, developmental stages, sexes and all sample, respectively. **(E**–**H)** The pairwise variations V_n_/V_n+ 1_ of candidate genes in four treatments mentioned above.

Two parameters of the M values and the pairwise variation V were calculated by geNorm software. The lower M values indicate that the gene was expressed more stably. The M values of twelve candidate genes were lower than 1.5 in different treatments, indicated that the expression levels of all genes were relatively stable. *RPL13A* and *RPS3A* were ranked as the most stable reference genes among in different tissues, sexes and all samples, which with the minimum M value 0.260, 0.054 and 0.308, respectively (Fig. 2A,C,D). However, *EF1α* and *EIF4A* were recommended as the most stable genes in different developmental stages, and the M value of the two genes were both at 0.135 (Fig. 2B). The role of pairwise variation V is determines the optimal number of reference genes for normalization. As shown in Fig. 2E–H, the pairwise variations V_2_/V_3_ were less than 0.15 in four different treatments, including tissues, developmental stages, sexes and all samples, suggesting that the optimal number of reference genes for normalization would be two. In addition, the least stable reference genes were *β-Tubulin1* (M value = 0.819) in the case of tissues, *β-Tubulin2* (M value = 0.786, 1.245) in different developmental stages and sexes, and *Actin* (M value = 1.425) in the case of all samples.

#### NormFinder analysis

**Figure 3:**
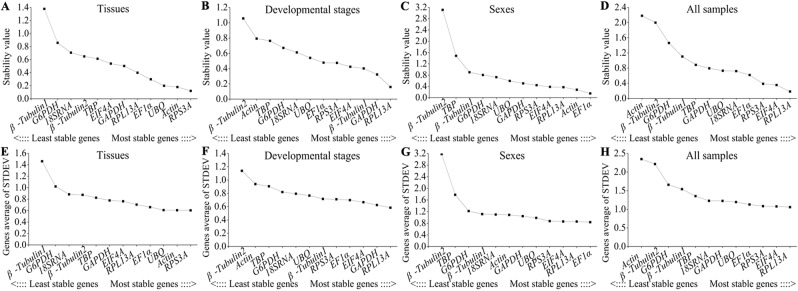
Stability analyses of candidate reference genes by NormFinder and Delta Ct algorithms. **(A**–**D)** The stability values of candidate genes calculated by NormFinder software in tissues, developmental stages, sexes and all sample, respectively. **(E**–**H)** The average STDEV values of candidate genes calculated by Delta Ct algorithms in four treatments mentioned above.

The optimal normalization gene among a set of candidate genes was identified by NormFinder software, providing a stability value of each genes that was negatively correlated with their stability. Based on the rank order assigned by NormFinder, *RPL13A* (stability value = 0.159, 0.321) was the best independent gene in different developmental stages and all samples, whereas *RPS3A* (stability value = 0.121) and *EF1α* (stability value = 0.150) were the optimal reference genes in different tissues and sexes, respectively (Fig. 3A–D). Similar to geNorm analysis, *β-Tubulin1*, *β-Tubulin2* and *Actin* with maximum stability values (1.379, 1.057, 3.112 and 2.181) were found to be the least stable reference genes in four treatments (Fig. 3A–D).

#### Delta Ct analysis

The Delta Ct algorithm is similar to geNorm, which also relies on the relative pairwise comparisons. The average standard deviation (SD) value of each gene was inversely proportional to the stability. The results showed that the most and least stable genes in four treatments recommended by Delta Ct were in accordance with the analysis by NormFinder (Fig. 3A–C,E,F). In all samples, *RPL13A* with the lowest SD value at 1.058 was ranked as the most stable gene, followed by *EIF4A* (1.076), *RPS3A* (1.085), *EF1α* (1.128), *UBQ* (1.194), *GAPDH* (1.224), *18SRNA* (1.227), *TBP* (1.354), *β-Tubulin1* (1.541), *G6PDH* (1.659), *β-Tubulin2* (2.215) and *Actin* (2.348) (Fig. 3H).

#### BestKeeper analysis

**Figure 4:**
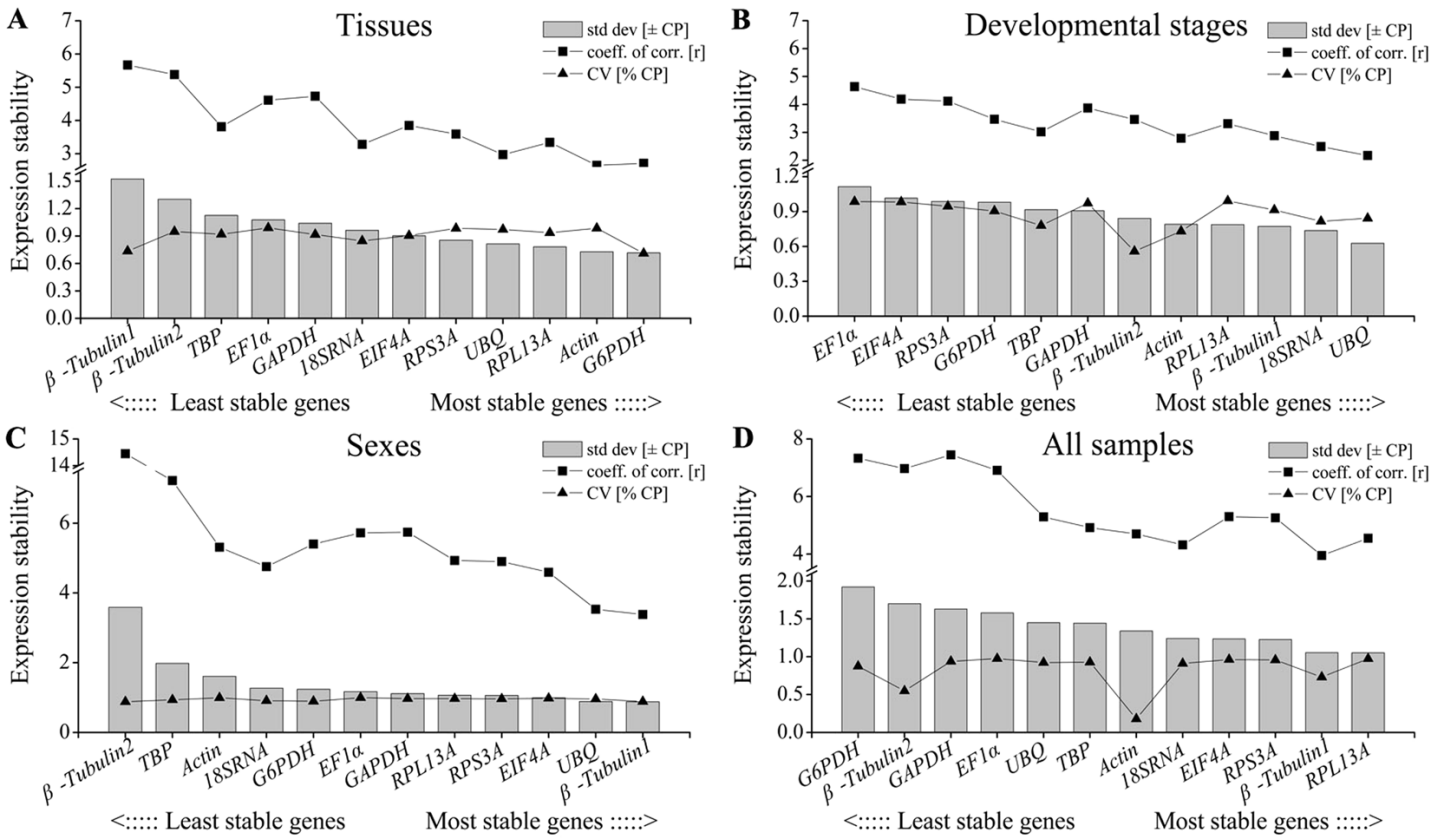
Expression stability of candidate reference genes in four experimental groups calculated by Bestkeeper.

BestKeeper program is used to estimate three major parameters of each candidate gene from raw Ct values, including the SD value, coefficient of variation (CV) and correlation coefficient (r), and the more stable genes usually have the lower SD and CV values, and higher r value. The results showed that *G6PDH* (SD value = 0.716), UBQ (SD value = 0.628), *β-Tubulin1* (SD value = 0.880) and *RPL13A* (SD value = 1.053) with the lowest SD values were recommended as the most stable reference genes in tissues, developmental stages, sexes and all samples, respectively (Fig. 4). Interestingly, *G6PDH* and *β-Tubulin1* expression showed significant variation, which were ranked as least reference gene in all samples and tissues although they were the best genes in tissue and sex treatments, respectively (Fig. 4).

#### Comprehensive ranking of RefFinder

**Figure 5:**
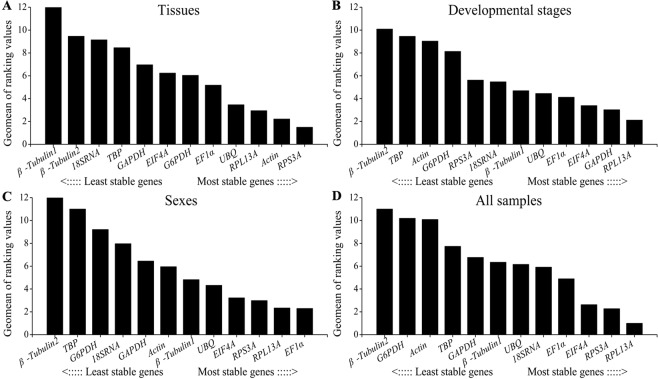
Comprehensive stability of candidate reference genes in four experimental groups determined by RefFinder.

RefFinder is a comprehensive web-based program that integrates all four above- mentioned software tools to rank the stability of candidate reference genes based on the Geomean of ranking values. The comprehensive rankings of candidate genes in four treatments were showed in Fig. 5. In tissue, developmental stage and sexes samples, *RPS3A*, *RPL13A* and *EF1α* were the most desired reference genes and followed with *Actin*, *GAPDH* and *RPL13A*, respectively (Fig. 5A–C). In all samples, the overall ranking followed by *RPL13A*, *RPS3A*, *EIF4A*, *EF1α*, *18SRNA*, *UBQ*, *β-Tubulin1*, *GAPDH*, *TBP*, *Actin*, *G6PDH* and *β-Tubulin2* (Fig. 5D). In addition, the stability rankings of all candidate genes by five analysis software were showed in Table 2.

**Table 2:** Expression stability ranking of the twelve candidate reference genes.

Experimental conditions	Methods	Stability ranking											
		1	2	3	4	5	6	7	8	9	10	11	12
Tissues	geNorm	*RPL13A/RPS3A*	*Actin*	*UBQ*	*EF1α*	*EIF4A*	*GAPDH*	*TBP*	*18SRNA*	*β-Tubulin2*	*G6PDH*	*β-Tubulin1*	
	NormFinder	*RPS3A*	*Actin*	*UBQ*	*EF1α*	*RPL13A*	*GAPDH*	*EIF4A*	*TBP*	*β-Tubulin2*	*18SRNA*	*G6PDH*	*β-Tubulin1*
	Delta Ct	*RPS3A*	*Actin*	*UBQ*	*EF1α*	*RPL13A*	*EIF4A*	*GAPDH*	*TBP*	*β-Tubulin2*	*18SRNA*	*G6PDH*	*β-Tubulin1*
	Bestkeeper	*G6PDH*	*Actin*	*RPL13A*	*UBQ*	*RPS3A*	*EIF4A*	*18SRNA*	*GAPDH*	*EF1α*	*TBP*	*β-Tubulin2*	*β-Tubulin1*
	RefFinder	*RPS3A*	*Actin*	*RPL13A*	*UBQ*	*EF1α*	*G6PDH*	*EIF4A*	*GAPDH*	*TBP*	*18SRNA*	*β-Tubulin2*	*β-Tubulin1*
Developmental stages	geNorm	*EF1α/EIF4A*	*GAPDH*	*RPS3A*	*RPL13A*	*G6PDH*	*β-Tubulin1*	*UBQ*	*18SRNA*	*TBP*	*Actin*	*β-Tubulin2*	
	NormFinder	*RPL13A*	*GAPDH*	*β-Tubulin1*	*EIF4A*	*RPS3A*	*EF1α*	*UBQ*	*18SRNA*	*G6PDH*	*TBP*	*Actin*	*β-Tubulin2*
	Delta Ct	*RPL13A*	*GAPDH*	*EIF4A*	*EF1α*	*RPS3A*	*β-Tubulin1*	*UBQ*	*18SRNA*	*G6PDH*	*TBP*	*Actin*	*β-Tubulin2*
	Bestkeeper	*UBQ*	*18SRNA*	*β-Tubulin1*	*RPL13A*	*Actin*	*β-Tubulin2*	*GAPDH*	*TBP*	*G6PDH*	*RPS3A*	*EIF4A*	*EF1α*
	RefFinder	*RPL13A*	*GAPDH*	*EIF4A*	*EF1α*	*UBQ*	*β-Tubulin1*	*18SRNA*	*RPS3A*	*G6PDH*	*Actin*	*TBP*	*β-Tubulin2*
Sexes	geNorm	*RPL13A/RPS3A*	*EIF4A*	*EF1α*	*UBQ*	*β-Tubulin1*	*18SRNA*	*GAPDH*	*Actin*	*G6PDH*	*TBP*	*β-Tubulin2*	
	NormFinder	*EF1α*	*Actin*	*RPL13A*	*EIF4A*	*RPS3A*	*GAPDH*	*UBQ*	*18SRNA*	*G6PDH*	*β-Tubulin1*	*TBP*	*β-Tubulin2*
	Delta Ct	*EF1α*	*RPL13A*	*EIF4A*	*RPS3A*	*UBQ*	*GAPDH*	*Actin*	*18SRNA*	*β-Tubulin1*	*G6PDH*	*TBP*	*β-Tubulin2*
	Bestkeeper	*β-Tubulin1*	*UBQ*	*EIF4A*	*RPS3A*	*RPL13A*	*GAPDH*	*EF1α*	*G6PDH*	*18SRNA*	*Actin*	*TBP*	*β-Tubulin2*
	RefFinder	*EF1α*	*RPL13A*	*RPS3A*	*EIF4A*	*UBQ*	*β-Tubulin1*	*Actin*	*GAPDH*	*18SRNA*	*G6PDH*	*TBP*	*β-Tubulin2*
All samples	geNorm	*RPL13A/RPS3A*	*EIF4A*	*EF1α*	*GAPDH*	*UBQ*	*18SRNA*	*TBP*	*G6PDH*	*β-Tubulin1*	*β-Tubulin2*	*Actin*	
	NormFinder	*RPL13A*	*EIF4A*	*RPS3A*	*EF1α*	*18SRNA*	*UBQ*	*GAPDH*	*TBP*	*β-Tubulin1*	*G6PDH*	*β-Tubulin2*	*Actin*
	Delta Ct	*RPL13A*	*EIF4A*	*RPS3A*	*EF1α*	*UBQ*	*GAPDH*	*18SRNA*	*TBP*	*β-Tubulin1*	*G6PDH*	*β-Tubulin2*	*Actin*
	Bestkeeper	*RPL13A*	*β-Tubulin1*	*RPS3A*	*EIF4A*	*18SRNA*	*Actin*	*TBP*	*UBQ*	*EF1α*	*GAPDH*	*β-Tubulin2*	*G6PDH*
	RefFinder	*RPL13A*	*RPS3A*	*EIF4A*	*EF1α*	*18SRNA*	*UBQ*	*β-Tubulin1*	*GAPDH*	*TBP*	*Actin*	*G6PDH*	*β-Tubulin2*

### Validation of recommended candidate reference genes

**Figure 6:**
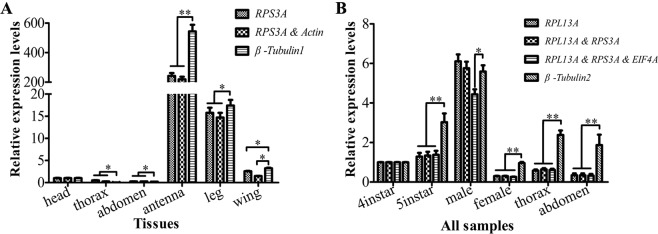
Validation of the gene stability measures. Expression levels of a target gene, *OBP3*, in six tissues (**A**) and all samples (including two tissues, two developmental stages, and two sexes) (**B**) were tested by different normalization reference genes. Bars represent the means and standard deviations of three biological replicates. Single asterisk indicates significant differences among different treatment within *OBP3* expression by normalized by reference gene or combination (P < 0.05, Duncan’s test), and double asterisks show extremely significant differences in the expression of *OBP3* (P < 0.01, Duncan’s test).

To validate the suitability of recommended reference genes, the mRNA expression of target gene *OBP3* in six tissues and all samples (including two tissues, two developmental stages and two sexes) was quantified by the best and worst candidate genes (or gene combinations). Among six tissues, the normalized expression results of *OBP3* were similar to those determined using *RPS3A* (the best candidate gene) and the combination of *RPS3A*/*Actin* (the best two), respectively. However, the expression levels of *OBP3* normalized using *β-Tubulin1* were significantly higher than using *RPS3A* or combination of *RPS3A*/*Actin* in three tissues (antenna, leg and wing) and lower in two tissues (thorax and abdomen) (Fig. 6A). Similarly, the expression of *OBP3* quantified by *β-Tubulin2* (the worst gene) were significantly greater than by the combination of *RPL13A*/*RPS3A* (the best two genes) or *RPL13A*/*RPS3A*/*EIF4A* (the best three genes) in all samples except male adult (Fig. 6B).

## Discussion

qRT-PCR is one kind of conventional method that can quantify the expression of nucleic acidin genetic researches. Evaluate appropriate reference genes should be the key link to get rid of differences and obtain the exact qRT-PCR results^15^. However, it is difficult to apply a universal appropriate reference gene for different experimental samples. Terribly, the true consequences of target gene’s expression would be concealed if using the bad reference genes^28,29^. Hence, in order to ensure stable expression of reference genes in a given species, all candidate genes should be assessed under a certain treatments^6,30,31^. In our study, the stability of twelve candidate genes was identified by five algorithms under multiple tissue, developmental stage and sex treatments in *H*. *theivora*. So far, no suitable reference genes are available for this insect or the genus of *Helopeltis*.

Our results confirmed that the stability of reference genes was variable dramatically under different treatments. For example, *Actin* was stably expressed under the tissue-specific conditions in *H*. *theivora*; but its expression varies significantly among the rest of samples (Table 2). This is similar to the results from Mustard aphid, *L*. *erysimi* (Hemiptera: Aphididae), in which the expression of *Actin* was stable under artificial diet treatments, but variable across different developmental stages^22^. Meanwhile, the results of different softwares can vary based on different algorithms, which are consistent with the previous results of other insects^32,33^. For instance, under developmental stage samples in *H*. *theivora*, *EF1α* and *EIF4A* were recommended as internal standard by geNorm, *RPL13A* was rated as the best gene by both NormFinder and Delta Ct, whereas *UBQ* was top choice by BestKeeper (Table 2). Therefore, the combined use of these algorithms can ensure the results are more credible. To this end, RefFinder was used to calculate the integrated ranking of stability for all candidate reference genes.

In recent years, increasing researches have indicated that a single reference gene used for the normalization in qRT-PCR may influence the accuracy of expression level^6,15^. Analysis of relative gene expression utilizing multiple reference genes showed up as a more reliable alternative^34–36^. Generally, geNorm is used to determine the optimal number of reference genes^6,7^. By focusing our findings, according to analysis of the minimum number V by geNorm, all treatments including tissues, developmental stages, sexes and all samples were suitable for using two reference genes.

In this study, we discovered two types of candidate genes (proteins associated with ribosome and protein production) showed relative stability in most samples in *H*. *theivora*, which were widely used for qRT-PCR in several species or treatments. Eukaryotic ribosome, consisting of four rRNAs and more than eighty ribosomal proteins, is one of the most evolutionarily conserved organelles^37^. Ribosomal proteins participates in the process of ribosome synthesis, transcription and cell development regulation, cell differentiation and rRNA processing, etc^38^. Previous study showed that some ribosome associated genes have been used as stable internal reference for quantitative analysis. Shu *et al*. (2018) evaluated eight candidate reference genes for relative quantification in *Spodoptera litura*, and the results indicated that *RPL13A* and *RPL7A* were the best genes in larva and fat body samples, respectively. *RPL13A* was also considered to be stable for tissues, developmental stages and sexes in *Aphidius gifuensis* and for low-temperature treatments in *Thitarodes armoricanus*^39,40^. Other ribosomal proteins have also been identified as internal control, such as *RPS18* for the gene expression associated with seasonal variation and division of labor in *Apis mellifera*^41^, *RPS11* for heat stress conditions in *Chilo suppressalis* (Walker)^42^, *RP49* for developmental stages in *A*. *mellifera*^43^, *RPL32* and *RPS15* for tissue samples in *H*. *armigera*^12^, *RPL7A* and *RP18* for most tissues in *Lethrus apterus*^11^, and so on. Similarly, our results demonstrated that *RPL13A* and *RPS3A* were consistently stably expressed throughout a majority of experimental samples in *H*. *theivora*.

The conserved nuclear gene *EF1α*, a G-protein that binds and recruits aa-tRNAs to the A-site of the ribosome, has been valuable as a higher-level phylogenetic marker in insects and it has also been widely used for stable reference gene^44,45^. For example, *EF1α* was the relatively stable gene for developmental stages and photoperiods in *Harmonia axyridis*^33^, for cuticle in *S*. *litura*^15^, for developmental stages in *Coleomegilla maculate*^32^, and for RNAi treatments in *Coccinella septempunctata*^9^. In the present study, consistent with above results, *EF1α* was ranked as the most stable gene in sex samples of *H*. *theivora*. Interestingly, those structure and metabolism related proteins (*Actin*, *Tubulin*, *GAPDH* and *G6PDH*) were ranked as less stable genes in this study, though these genes have been commonly used as internal controls in many expression researches^13,46–48^.

In summary, this is the first report of reference genes expression in *H*. *theivora*. The expression profiles of twelve candidate reference genes under three treatments (different tissues, developmental stages and sexes) were investigated by five algorithms, geNorm, NormFinder, BestKeeper, Delta Ct and RefFinder. A pair of stable reference genes was particularly recommended for each experimental condition as follows: *RPS3A* and *Actin* for tissues, *RPL13A* and *GAPDH* for developmental stages, *EF1α* and *RPL13A* in sex samples, and *RPL13A* and *RPS3A* across all samples (three treatments). Our results demonstrate that there is no a universal reference gene can suitable for all experimental conditions. In addition, this study represents the critical first step to normalize qRT-PCR data for the functional genomics research in *H*. *theivora*.

## Materials and Methods

### Insect rearing

*H*. *theivora* adults were collected from cocoa pod in the field of Spice and Beverage Research Institute, Chinese Academy of Tropical Agricultural Sciences, and reared on green beans and 10% sucrose solution in laboratory. A laboratory colony was established and maintained at 26 ± 1 °C, 75 ± 5% relative humidity and 14:10 light: dark cycle. Sample collection begins with the second generation insect.

### Sample collection

Individuals at six developmental stages were collected, including 1^st^ instar nymphs (30 individuals), 2^nd^ instar nymphs (20 individuals), 3^rd^ instar nymphs (15 individuals), 4^th^ instar nymphs (10 individuals), 5^th^ instar nymphs (5 individuals) and adults (whole body, 3 individuals). The six tissues of adults (10∼15 individuals), including antenna, head, thorax, abdomen, leg and wing, were dissected by scalpel and washed in cold phosphate buffered saline. The four sex samples were also collected and dissected, including adult (adultF and adultM, 3 individuals) and thorax (thoraxF and thoraxM, 10 individuals) of both female and male. All samples were placed in 1.5 ml centrifuge tubes (50∼100 mg each tube), flash frozen in liquid nitrogen and kept in −80 °C successively. Each treatment contained three biological replications.

### Total RNA isolation and cDNA synthesis

Total RNA from different tissues, developmental stages and sexes were extracted by TRIzol Reagent (Invitrogen, USA) following the manufacturer’s protocol. Each sample was disrupted and homogenized with 1 ml TRIzol reagent. Depending on the pellet size, moderate amounts of RNase-free water was added to dissolve the precipitate. The concentration and purity of total RNA were measured by fluorescence microplate reader (BioTek, USA). The isolated RNA (1 µg per samples) was reversely transcribed to first-strand cDNA using PrimeScript RT reagent Kit with gDNA Eraser (TaKaRa, China) following the manufacturer’s instructions. The products were stored at −20 °C.

### Real-time PCR (RT-PCR) and quantitative real-time PCR (qRT-PCR)

Twelve candidate reference genes were identified and cloned based on the transcriptome data of *H*. *theivora*. The primers and sequence data of twelve genes were listed in Table 1 and S1. RT-PCR amplifications were performed by denaturing at 94 °C for 3 min, followed by 35 cycles of 94 °C for 30 s, 60 °C for 30 s and 72 °C for 1 min, with a final extension at 72 °C for 10 min. The amplification products were detected by 1% agarose gel electrophoresis and extracted by E.Z.N.A.^TM^ Gel Extraction Kit (Omega, USA). The DNA fragments were ligated with pMD-19T and transformed into *Escherichia coli* DH5α (TaKaRa, China). Plasmid were extracted by E.Z.N.A.^TM^ Plasmid Miniprep Kit II (Omega, USA) and used as the templates for standard curve of candidate genes.

qRT-PCR reactions were performed with three technical replicates on BioRad CFX96 Real-Time PCR detection system using 2× TB Green Premix Ex Taq (TaKaRa, China). Amplification conditions consisted of an initial denaturation at 95 °C for 3 min, followed by 40 cycles of 95 °C for 10 s and 60 °C for 30 s. After reaction, a melting curve analysis from 65 °C to 95 °C was applied to all reactions to ensure consistency and specificity of the amplified product. A series of 10-fold dilution of plasmids were used to create the five-point standard curves using the linear regression model^18^. The regression equation was carried out to calculate the efficiency (E) and correlation coefficient (*R*^2^) of each primer pair. The efficiencies (E) of corresponding primers were estimated according to the equation:

$${\rm{E}}=({10}^{[-1/{\rm{slop}}e]}-1)\times 100.$$

### Data mining and statistical analysis

The cycle threshold values (Ct values) from qRT-PCR were collected and the stability of candidate reference genes were ranked by using Microsoft Excel based software tools (or algorithm), including geNorm, NormFinder, BestKeeper, Delta Ct and RefFinder. The relative quantities converted from the raw Ct values (the highest relative quantity of gene was set to 1) were used as input data for geNorm and NormFinder. geNorm calculates the expression stability value (M) and pair wise variation (V). Gene expression is considered stable when the M value is below 1.5, and the lowest M values are produced by genes with the most stable expression. Besides, the value of V_n_/V_n+1_ was used for determine the optimal normalization reference gene number and geNorm V_n_/V_n+1_ < 0.15 could be the standard for better normalization^7^. NormFinder provides a stability value for each gene, which is a direct measure of the estimated both intra- and inter- group expression variation^17^. BestKeeper use raw Ct values and PCR efficiency to determine the optimal reference genes^18^. Delta Ct algorithm evaluated the stability rank order of reference genes by comparing the relative expression of pairwise genes within each sample^49^. Finally, the web-based program RefFinder (http://www.leonxie.com/referencegene.php), which is a comprehensive platform integrating above four algorithms, provided an overall ranking of the stability of candidate reference genes^50^.

Odorant binding protein-3 (*OBP3*, Accession number: MN062611) gene was used to evaluate the validity of selected reference genes under different experimental conditions. qRT-PCR amplification of *OBP3* was obtained from the transcriptome data and performed with primers: Forward (CAGCGGAGTCGAAGATACCC) and Reverse (TGGCCTTCGCTAGTTTTGGT). The relative expression levels of *OBP3* were determined according to the Ct values based on the 2^−ΔΔCt^ method^51^. All the treatments were performed in three biological and technical replicates, respectively. One-way ANOVA was used to compare the effects of treatments. Statistical analyses were performed using SPSS 20.0 (SPSS, Inc., USA).

## Supplementary information

Supplementary Figures and Tables

## Data Availability

All data generated or analyzed during this study are included in this published article and its Supplementary Information files.
