# Supplementary material for: Evaluation and Validation of Reference Genes for Quantitative Real-Time PCR in Helopeltis theivora Waterhouse (Hemiptera: Miridae)
Source: Sci Rep. 2019 Sep 16;9:13291. doi: 10.1038/s41598-019-49479-1 (PMC6746731; doi:10.1038/s41598-019-49479-1)
Supplement: Supplementary file 1 — Supplementary Figures and Tables [file 41598_2019_49479_MOESM1_ESM.doc]

**Evaluation and Validation of Reference Genes for Quantitative Real-Time PCR in** ***Helopeltis theivora* Waterhouse (Hemiptera: Miridae)**

Zheng Wang^1, †^, Qianqian Meng^1, †^, Xi Zhu^1, 2^, Shiwei Sun^1^, Shengfeng Gao^1^, Yafeng Gou^1^ & Aiqin Liu^1, 2*^

**Supplementary Information:**


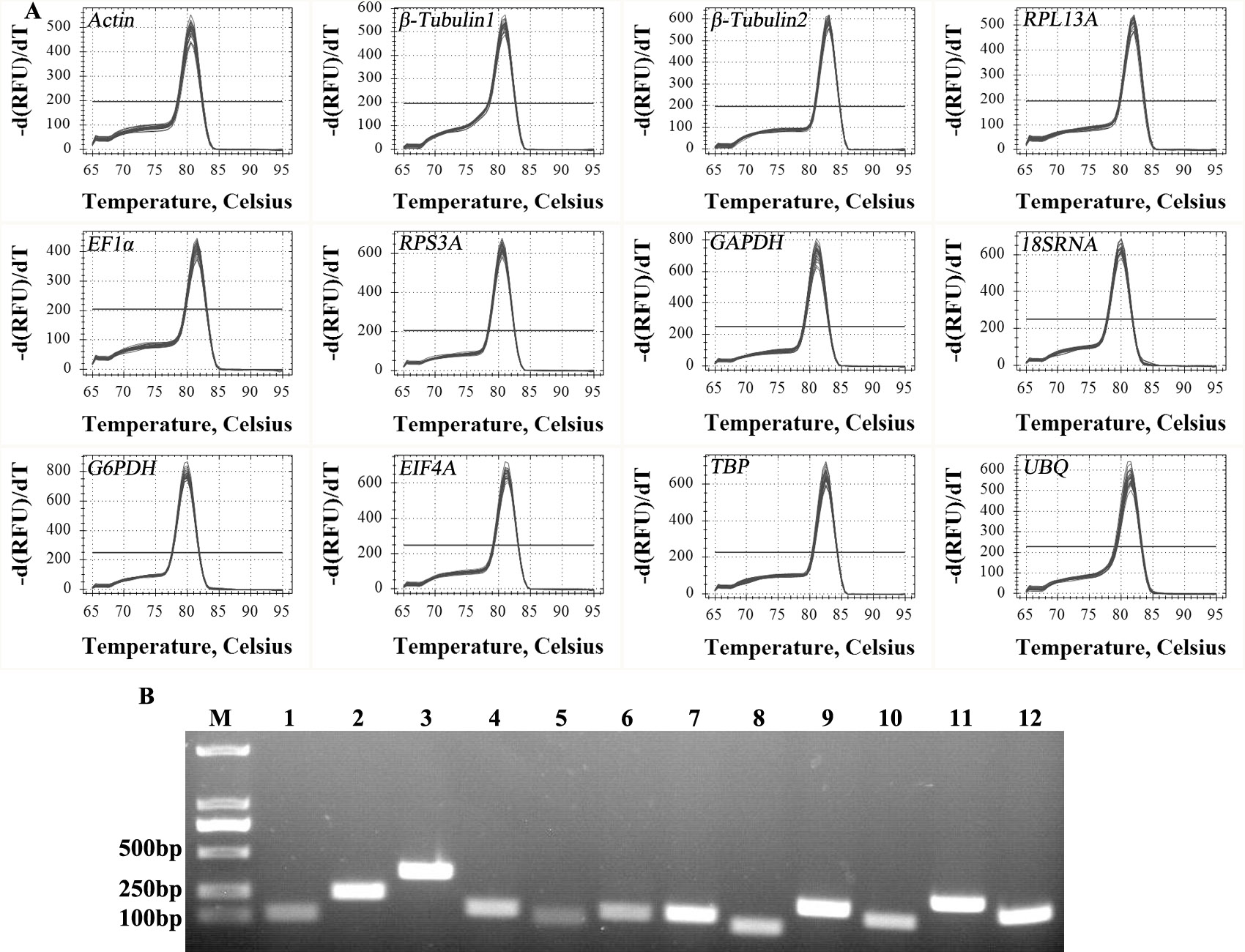


**Supplementary Figure 1.** Amplification specificity of candidate reference gene primers in qRT-PCR and RT-PCR. **(A)** Melt curve analysis of twelve candidate genes across sixteen samples (including six tissues, six developmental stages and four sexes, respectively). **(B)** Cropped agarose gel of twelve candidate genes PCR products. Lane M, DNA 2000 Marker, lane 1-12, *Actin*, *β-Tubulin1*, *β-Tubulin2*, *RPL13A*, *EF1*α, *RPS3A*, *GAPDH*, *18SRNA*, *G6PDH*, *EIF4A*, *TBP* and *UBQ*. Full-length gel is presented in Supplementary Figure 2.


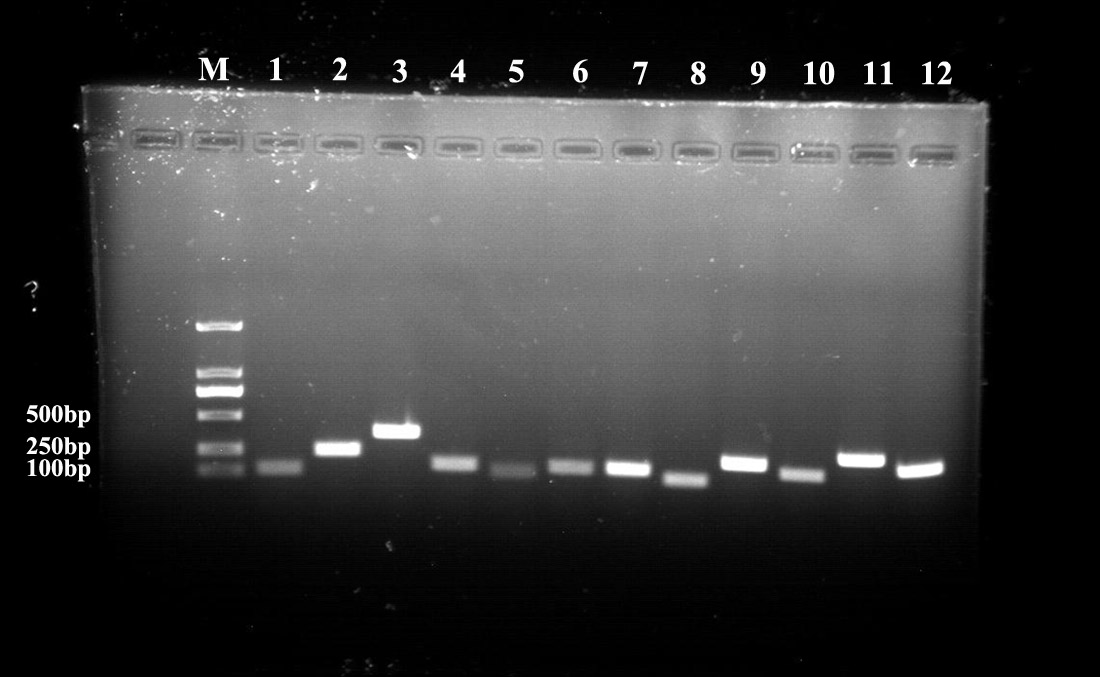


**Supplementary Figure 2.** Full-length gel of twelve candidate genes PCR products. Lane M, DNA 2000 Marker, lane 1-12, *Actin*, *β-Tubulin1*, *β-Tubulin2*, *RPL13A*, *EF1*α, *RPS3A*, *GAPDH*, *18SRNA*, *G6PDH*, *EIF4A*, *TBP* and *UBQ*.

**Supplementary Table 1. Sequence data of twelve candidate genes and one target gene**

| **Gene** | **Length (bp)** | **Partial sequence** |
| --- | --- | --- |
| **Candidate reference genes** | | |
| *Actin* | 468 | ATGACACAGATCATGTTCGAGACCTTCAACACACCCGCCATGTACGTGGCCATCCAGGCTGTGCTGTCCCTGTACGCGTCCGGTCGTACCACCGGTATCGTGCTGGACTCCGGCGACGGTGTGTCCCACACAGTGCCCATCTACGAGGGCTACGCCCTGCCGCACGCCATCCTCCGTCTGGACTTGGCCGGCCGTGACCTCACAGACTACCTGATGAAGATCCTCACCGAGCGTGGTTACTCATTCACCACCACCGCTGAGCGTGAAATCGTCCGTGACATCAAGGAGAAGCTCTGCTATGTCGCCCTCGACTTCGAGCAGGAGATGGCCACCGCCGCCGCCTCCACCTCCCTCGAGAAGTCCTACGAGCTTCCCGACGGTCAGGTCATCACCATCGGTAACGAGAGGTTCCGTTGCCCCGAAGCCCTGTTCCAGCCTTCCTTCCTGGGTATGGAATCTTGCGGTATC |
| *β-Tubulin1* | 1344 | ATGAGAGAAATCGTTCACCTTCAGGCTGGACAGTGCGGTAACCAAATCGGAGCCAAATTTTGGGAAATTATTTCTGACGAACATGGTATAGATCCTACTGGCGCTTATCATGGTGATTCCGATCTCCAGCTTGAAAGGATCAATGTATATTACAATGAAGCATCAGGTAATGGCAACTATGTACCTCGAGCGATTCTCGTCGATTTGGAACCTGGCACAATGGATTCTGTTCGTTCTGGACCTTTTGGCCAAATTTTCAGACCAGACAATTTCGTATTTGGTCAGTCAGGCGCTGGCAACAATTGGGCTAAGGGACATTACACCGAAGGAGCTGAGTTGGTCGATGCAGTTTTAGATGTAGTTCGAAAGGAAGCCGAAGGTTGCGACTGCTTGCAGGGATTCCAATTGACTCATTCTCTCGGAGGCGGTACCGGATCTGGAATGGGAACCCTTCTTATCTCCAAAATTCGAGAAGAATATCCTGATCGTATCATGAACACTTACTCTGTCGTACCATCTCCCAAGGTGTCAGATACAGTAGTTGAACCATACAATGCCACTCTCTCTGTTCACCAACTTGTTGAAAACACTGACGAAACCTACTGTATTGACAATGAAGCTTTGTATGACATTTGTTTCCGCACATTGAAGCTTTCAACTCCCACTTATGGTGATCTTAATCATTTGGTTTCGTTAACTATGTCTGGAGTCACCACTTGTCTGAGATTCCCAGGTCAGTTGAACGCCGATCTTCGAAAATTGGCCGTCAATATGGTTCCATTCCCTCGACTTCACTTCTTCATGCCAGGTTTTGCACCACTCACCTCACGCGGATCTCAGCAATATCGAGCGCTCTCAGTTCCAGAGCTAACTGCTCAAATGTTTGACTCCAAAAACATGATGGCTGCCTGTGATCCTCGCCACGGACGTTACCTTACAGTTGCTGCTATTTTCAGGGGACGAATGTCGATGAAGGAAGTTGACGAGCAAATGCTTAATATTCAGAACAAAAATTCTTCGTATTTCGTCGAATGGATTCCCAACAATGTTAAGACGGCTGTGTGCGACATTCCACCGCGAGGACTGAAAATGTCAGCCACTTTCATCGGAAATTCTACTGCCATTCAGGAGCTGTTCAAGAGAATTTCTGAGCAATTCACTGCCATGTTCCGTCGAAAGGCTTTCCTCCATTGGTATACTGGTGAAGGTATGGACGAAATGGAATTCACCGAAGCCGAGTCGAACATGAACGATTTGGTTTCTGAGTATCAACAATACCAAGAGGCCACTGCGGACGAAGACGCCGAATTCGACGAAGAACAAGAGCAAGAAGTAGAAAATTAA |
| *β-Tubulin2* | 1350 | ATGAGGGAAATCGTGCACATTCAAGCCGGCCAATGCGGCAACCAAATCGGAGCTAAGTTTTGGGAAATCATTTCCGATGAACACGGTATCGACCCGACCGGAGCCTATCACGGCGATTCTGACCTCCAACTCGAAAGGATCAACGTCTACTATAACGAAGCTTCAGGAAAAGGAGGCGGCAAATATGTTCCCCGCGCCATTTTGGTTGATCTGGAACCCGGCACAATGGATTCCGTCAGGTCCGGCCCATTCGGTCAAATATTTAGGCCCGACAATTTCGTTTTTGGACAATCCGGCGCTGGTAATAACTGGGCTAAAGGACATTACACCGAAGGTGCTGAATTAGTCGATTCCGTCCTCGATGTCGTTAGGAAAGAAGCTGAAAGCTGCGATTGCCTACAAGGATTCCAGCTTACTCATTCGCTCGGAGGTGGCACTGGATCCGGTATGGGCACTTTACTCATTTCGAAAATCCGCGAAGAATACCCAGACAGGATCATGAACACCTACTCTGTCGTTCCCTCGCCAAAGGTCTCGGATACTGTCGTCGAACCGTACAACGCCACACTTTCCGTCCATCAACTCGTCGAAAACACGGACGAAACATACTGTATTGACAACGAAGCTCTCTACGACATCTGTTTCAGAACGCTCAAACTTTCAACCCCCACATACGGCGATCTTAACCATCTCGTATCACTCACCATGTCGGGCGTCACCACCTGCCTTAGGTTCCCTGGTCAATTGAATGCCGATCTCCGTAAATTGGCCGTCAACATGGTTCCCTTCCCACGTCTCCACTTCTTCATGCCTGGATTCGCTCCACTCACGTCAAGAGGATCTCAACAGTACAGAGCTCTCACTGTGCCAGAACTGACTCAGCAAATGTTCGACGCCAAGAACATGATGGCCGCCTGCGACCCAAGACACGGACGCTACCTCACAGTCGCCGCTATTTTCAGAGGGCGCATGTCGATGAAAGAAGTCGACGAACAAATGTTGAACATTCAAAACAAAAATAGCTCCTACTTCGTCGAATGGATTCCCAATAACGTTAAAACCGCCGTCTGCGACATCCCGCCCCGAGGTCTCAAAATGGCAGCCACTTTCATCGGAAATTCGACCGCCATTCAAGAATTATTCAAGAGAATTTCCGAACAATTTACCGCCATGTTCAGGCGAAAGGCTTTCTTGCATTGGTACACCGGAGAAGGTATGGATGAAATGGAATTCACCGAAGCCGAATCAAACATGAACGATCTTGTATCAGAATATCAGCAGTACCAGGAAGCGACTGCTGACGAAGACGCCGAATTCGACGACCAAGAACACGAAGTTGACGAAAACTAA |
| *RPL13A* | 615 | ATGACGGGGTTCAGCGACAAGCCTTTGCTTATTGACGGCAAAGGCCACCTTTTGGGCCGTTTGGCTTCCATCGTGGCCAAAGCTGCTCTCAACGGCAACAGAGTCGTTGTTGTCAGATGTGAACAAATCAACATTTCTGGAAATTTCTTCAGGAGTAAATTGAAGTATTTGGCGTTCTTGAGGAAACGTTGCAATGTCAACCCAGCTCGAGGCCCCTTCCATTTCAGAGCTCCAAGCAAAATTCTTCACAGAACCATCCGAGGTATGGTTCCACACAAAATCGAAAGGGGAAAGGCCGCTTTGAGAAGGATAAAATTGTTCGAGGGGTGCCCGCCTCCTTACGACAAAAGGAAAAGGTTGGTTGTCCCCAGCGCTATGCGAGTCCTCTGTTTGAAACCAGGGCGTGCCTACTGTCATCTTGGTCGTCTTTCTGCCGAAGTCGGATGGAAATATAAGGACGTCGTCAGAGCTCTTGAAACCAAGAGGAAGGTTCGCGCTGTACTTGATATTCGCAAGAGAAACGGTCTTAAGAAACTGACTAAAAAGGCTGGAGAAAAAGTCAGCAAAGCTTCCGCTCAATATACGGCGGTCATCAACTCGTATGGATACCGATAA |
| *EF1α* | 1389 | ATGGGTAAAGAAAAGATTCATATTAACATCGTCGTCATCGGTCACGTCGACTCTGGTAAATCCACGACGACCGGACATTTGATCTACAAATGCGGTGGTATCGACAAACGTACGATCGAAAAATTCGAGAAGGAAGCCCAAGAAATGGGCAAAGGTTCCTTCAAGTACGCTTGGGTCTTGGACAAATTGAAGGCCGAACGTGAACGTGGTATCACCATCGATATTGCCCTGTGGAAGTTCGAAACTGCCAAATACTACGTAACCATCATTGATGCTCCCGGACACAGGGATTTCATCAAAAACATGATTACCGGAACCTCACAGGCCGATTGTGCTGTGTTGATCGTTGCTGCTGGTACTGGTGAATTCGAAGCTGGTATCTCCAAGAACGGACAAACTCGTGAGCACGCTCTCCTCGCTTTCACGCTTGGAGTCAAGCAGCTCATCGTTGGTGTCAACAAGATGGACTCCACTGAACCTGCTTACAGCGAGTCTCGATTCGAAGAAATCAAGAAGGAAGTCTCTTCTTACATCAAGAAGATCGGTTACAATCCCGCCGCTGTCGCTTTCGTTCCAATCTCTGGTTGGCACGGAGACAACATGTTGGAGCCCTCCGACAAGATGCCCTGGTTCAAGGGGTGGGCCATCGAAAGGAAAGAAGGCAAAGCCGACGGCAAATGCCTTATCGAAGCTTTGGACGCCATCTTGCCCCCGTCTCGTCCCACCGACAAGGCTTTGAGGCTTCCACTTCAGGATGTGTACAAAATCGGTGGTATTGGAACTGTCCCAGTCGGTCGTGTTGAGACTGGTGTTCTCAAGCCTGGTATGGTCGTCACTTTCGCCCCGGTCAACTTGACCACTGAAGTCAAGTCCGTTGAAATGCACCACGAAGCTTTGCAAGAAGCCGTGCCCGGAGACAACGTTGGTTTCAACGTCAAGAACGTCTCTGTCAAGGAATTGCGTCGTGGTTATGTCGCCGGTGATTCCAAAGCCTCTCCTCCTAAGGCTGCTGCTGATTTCACCGCTCAGGTTATTGTCCTCAACCATCCTGGACAAATCGCTAATGGGTACACTCCAGTTTTGGATTGCCACACTGCCCATATTGCTTGCAAATTCGCTGACATCAAAGAAAAATGCGACCGTCGTACTGGTCAGACCACTGAGCAGAACCCCAAGTCCATCAAGTCCGGTGACGCTGCTATCATCAATCTCATTCCGACTAAACCTATGTGCGTTGAATCCTTCCAGGAGTTCCCTCCTCTTGGACGTTTCGCCGTTCGTGACATGAGACAAACTGTTGCCGTAGGTGTTATCAAGAGCGTCACCAATAAAGACATTTCCACTGGCAAAGTAACGAAGGCCGCCGAAAAGGCCCAAAAGAAGAAATAA |
| *RPS3A* | 789 | ATGGCTGTCGGTAAAAATAAAGGGCTTTCCAAAGGAGGAAAGAAAGGAGTTAAGAAGAAGGTAGTGGATCCCTTCACCCGTAAAGATTGGTACGATGTCAAAGCTCCTTCAATGTTCAAGAAACGTCAGGTCGGCAAAACCCTCGTTAACCGAACTCAAGGCACAAAGATCGCCTCTGAAGGGTTAAAGGGCCGTGTTTTTGAAGTGTCTCTCGCCGACATTCAAGAAGACACCGATGCCGAAAGGTCGTTCAGAAAGTTCAGACTGATCGCCGAAGATGTTCAAGCTCGTAATGTCCTTACCAATTTCCACGGAATGGATTTGACAACAGACAAGCTTCGCAGCATGGTGAAAAAGTGGCAGACGTTGATTGAAGCCAACGTTGACGTCAAAACCACCGATGGCTACCTGTTGAGGGTTTTCTGCATAGGCTTCACCAACAAAGATCAGTTGTCTCAACGGAAAACTTGTTACGCTCAACACAACCAGGTTCGAGCGATTAGACGAAAAATGGTTGATGACATAACTTCAAACATTGTCAACATCGATTTGAAAGGAGTTGTTGAGAAATTGATCCCAGATTCAATCGCCAAGGACATTGAAAAACATTGTCAAGGCATCTATCCATTGCACGACGTCTACATCCGAAAGGTGAAAGTACTCAAGAAGCCGAGGTTCGAACTTAGCAAACTCCTCGAACTTCATGGAGATGGTAAAGGCAGTGATGAGCCTGGCGCTAAAGTAACGAGAACCGATGCCTACGAACCTCCAGTTCAGGAATCTGTTTAA |
| *GAPDH* | 1002 | ATGTCTAAAATTGGAATCAATGGATTCGGTCGTATCGGCCGCCTCGTTTTGAGGGCTTCCGTTGACAAAGGAGCCGAGGTGGTTGCCATCAATGACCCCTTTTTGGGCGTTGATTACATGGCCTACCTTTTGAAGTACGACTCAACTCACGGTCGCTTCAAGGGCAAGGTTGACATTGAAGGCGATTGTCTCGTTGTCAACGGAAAGAAAATCGCCGTTTTCCAAGAACGTGATCCCAAAGCCATCCCATGGGGCAAATCTGGCGCTGAATATGTCGTCGAATCGACTGGTGTTTTCACCACCATTGAAAAAGCCAAGGCTCATTTGGATGGAGGAGCTAAAAAAGTAATCATCTCCGCTCCATCAGCCGACGCGCCAATGTTCGTCGTTGGTGTCAATTTGGATGCTTACGATCCATCGATGACCGTCGTTTCAAACGCCTCTTGCACCACCAACTGCCTCGCTCCTTTGGCCAAAGTTATCGACGACAATTTCGGTATTGAAGAAGGCTTGATGACCACTGTTCACGCCGTCACAGCCACTCAAAAAACTGTCGACGGACCTTCTGGAAAGTTGTGGAGAGATGGACGTGGAGCCGGGCAAAACATCATTCCCGCCGCTACCGGTGCCGCTAAAGCTGTCGGCAAAGTCATCCCCAAGTTGAACGGCAAATTGACCGGTATGGCCTTCAGAGTGCCTGTACCCAACGTATCTGTCGTCGATCTCACAGTCAGGTTGTCCAAAGAGGCTACTTACGATGACATCAAGAAGAAGGTCAAGGAAGCGTCCGAAGGTCCTCTCAAGGGAATTTTGGGTTACACCGACGATCAAGTAGTAAGCGCCGATTTCATTGGAGACACCAACAGCTCGATTTTCGACGCTCAAGCGGGAATCCCATTGAACAACAAATTCGTGAAACTTGTTTCCTGGTATGACAACGAATTTGGATACTCCAACCGAGTTGTCGACCTCATCAAGTACATGCAAACCAAGGACAAATAA |
| *18SRNA* | 834 | ATGTCGGGAAGAAGGCCGGAACATCAGGCGCCTCCTGATATATTTTACAACGAGATCGAAGCACGAAAATACACTTCAAATTCTCGAATTATCGATATACAAGTGCAAATGAGCGAACGGGCTTTAGAATTACTGTGCCTTCCCGAAGACGAATCCTGTCTACTTCTCGATCTCGGATGCGGTTCAGGTTTGAGCGGTTCGGTTTTAGAAGAAAACGGTCACGTTTGGGTTGGAATGGACATTTCACAAGCGATGTTGAACGTTGCTGTCGAACGAGGAGTCGAAGGAGATTTAGTTTTGTCGGACCTTGGTCAAGGTGTACCTTTTCGAGCAGGTATGTTTGACGGAGCCATAAGCGTTTCCGCAATACAATGGTTGTGCAATTGCGACAAAAAGGATCACAACCCTGTCAAAAGGCTTGGCGCTTTTTTCTCATTGTTGTACACGTCGTTGTCTAGATGCGCCAGAGCTGTTTTTCAATTTTATCCGGAATCCCCGAGCCAGGTGGAGCTGATAACGACTCAAGCGACCAAAGCCGGTTTTTACGGCGGCCTCGTCGTTGATTATCCGAATTCGACAAAAGCGAAGAAATATTTTCTAGTTTTGATGACCGGACAAAGCGTCGCTCTTCCGAAAGCGTTGGGCGCTGAAAACGAAGAGGAATCTCGGGTTCAATACGGAAAACAAAGGTTAACGTATAAACAGATGAAAGGGAAGTCTTTGAAAAAGAGTAGAGATTGGATTTTGGAAAAGAAAGAACGAAGAAAACGACAAGGGTTGGAAGTGAGAGCGAATTCGAAATTCACAGGGAGAAGGCGATGCGGAAAACTTTGA |
| *G6PDH* | 1563 | ATGGGTCCATTTGTTCCGGATAATGTTGAGAGCAGCCTGACGCTCATCAGGAAGTCTCTTAGGAATCAAACCATAATGACGGAAGGAGCGATGTTCGAAGGCAATGCCTTCCATACGTTCATCATTATGGGAGCGTCGGGCGATCTGGCGAAAAAGAAAACTTATCCGACGGTTTGGTGGTTGTATCGCGACGGCCTGCTACCCAATAACATAGTCTTTGTCGGCTATGCTCGAAGTACTCTGACCATCGACGAATTGAAAGAACGCGTTCGACCTTACATGAAGGTACAACCTGACGAAGAACAAAAATACGAAGATTTTTGGAAATTGAACCATTACGTCAGCGGTACTTACACGTCGCGAACGGATTTTGAGCTTTTAAACCAAAAATTGTCGTCCCATGAGCCTGCGGCAGGTGCTAACAGAATTTTTTACCTTGCATTGCCTCCTTCTGTTTTTGAAATCGTAACCACAAATTTACGTAACACCTGCATGTCACCCAAGAGGTGGACCAGAATAATCATCGAAAAGCCCTTCGGCCGAGATTCAGATACCTCTCAAAGATTGTCCGATCATCTTGCCAGTTTATTCAAAGAAGAACAATTGTATCGTATCGACCATTATTTGGGAAAGGAAATGGTTCAAAACCTTATGATTCTGAGATTTGCCAATGCGATATTTAGCCCAACGTGGAATCGTCTGCATATAGCGTCCGTTCAAATTTCCTTCAAAGAGCCCTTCGGAACTGAAGGAAGAGGAGGTTATTTCAACGAATTCGGTATCATTCGAGACGTCATGCAAAATCATTTGATGCAAATACTTACTTTGGTCGCTATGGAAAAGCCAGCCTCGATCCATCCTGATGATATTCGCAATGAGAAAGTCAAAGTTCTCAAGTGCATCAAACCTCTACATGTGGACGACGTGGTTCTCGGCCAATATGTGGGAAATCCTGACGGCGAAGGGGACGCCAAAATTGGTTACTTGGACGATCCTACAGTGCCCAGTGGATCTACGACTCCCACGTTTTGTACAGCCATTCTGAAAATTGACAACGAACGTTGGGAAGGCGTTCCTTTCATTTTGAGATGCGGGAAAGCGTTGAACGAAAGGAAAGCCGAAATTAGGATTCAGTATCAGGACGTGCCCGGAGATATTTTTGGTGGAAAATGCAAAAGGAACGAACTAGTGATAAGAGTGCAACCCGGAGAAGCTGTTTACATCAAAATGATGACGAAAACGCCTGGAATGTCCTTTGACATGGAAGAAACCGAATTAGACCTCACCTACGGCAGTAGATACAAGGACGTTAAATTGCCAGACGCCTACGAACGCCTGATTCTTGATGTGTTCTGCGGTTCTCAAATGCACTTTGTGCGGTCAGACGAATTAGCTGAAGCTTGGAGGATATTCACTCCTCTTCTCCATCAATTAGAAATTGAAAATATTCGTCCCATCCCTTATGTGTACGGCTCGCGAGGACCCAACGAAGGCGATGAAATAACGAAAAACGCCAATTTCAAATATTATGGTTCCTACAAATGGGTTGAACCCTCCAAATAA |
| *EIF4A* | 1263 | ATGTCGGTAAATTCCTCAGACAAAAGAGAGGACTGGGACGATGGTTCGAAACATGCCGAGAAAGAGTCGCTTCATGGCGGCCCTCCCGGTATGGATCCTGACGGTATCATCGAGACCACATGGGATGAGGTTGTCGACAATTTTGACGACATGAACCTCAAAGAAGAACTCCTGAGAGGTATTTACGCTTACGGTTTTGAAAGACCTTCCGCTATTCAGCAACGCGCTATTATTCCCTGCATCAAGGGACTTGACGTCATTGCTCAGGCCCAATCAGGTACTGGCAAAACTGCTACATTTTCAATTTCCATACTTCAGCAGATTGATACTAACTTGCGAGAATGTCAAGCTCTTATTCTCGCTCCTACAAGAGAATTGGCCCAGCAGATCCAAAAAGTCGTTTACACTTTGGGAGATTTCATGGGCGCAATGTGCCACGCGTGTATCGGAGGCACCAACATTAGAGAAGACATACGTAAACTCGAGCAAGGAGTTCATGTTTTGGTCGGAACTCCGGGAAGAGTTTTAGATATGATCTCCAGACGAGCGTTGAAAACTAACACTATCAGGATATTCGTCCTAGATGAGGCCGATGAAATGTTGTCGCGAGGGTTCAAAGATCAAATCCACGATGTATTCAGAAGTCTTCCTCATCAAGTGCAGGTCATTTTACTTTCCGCAACAATCCCTAGTGACGTTCTTGAAGTCACTAATTGTTTTATGAGACAACCGATTCAGATTTTGGTGAAGAAAGAAGAGCTGACACTTGAAGGTATCAAACAATTTTACGTATCTGTAGAAAAAGAAGAGTGGAAACTTGATACCCTTTGCGATTTGTACGATACACTTAGTATCACTCAAGCTGTGATTTTCTGCAACACACGGCGTAAGGTCGACTGGCTTACTGAAAGTATGCACGGCCGTGATTTTACGGTGTCCGCTATGCATGGCGACATGGGTCAAAAAGAACGCGATTTGATTATGAAAGCGTTCAGAACTGGATCGAGCCGTGTTCTAATCACGACCGATCTTCTCGCTCGTGGTATCGATGTCCAACAAGTTTCCCTAGTTATCAACTACGATTTACCTTCGAATCTAGAAAATTACATTCACAGAATCGGGCGAGGTGGACGTTTTGGTCGTAAAGGAGTTGCCATTAATTTTGTCACTGAAGATGACAGAAAGACTCTCCAGGAAATTCAAAAATACTACAATACCGTCATAGAAGAAATGCCAATGAACGTTGCAGATTTATTCTACTAA |
| *TBP* | 879 | ATGGATCATATGCTTCCAAGTCCGGGATTCAGCATCCCCAGCATCGGTACTCCGTTGCACCAGCCGGAGGAAGATCAACAAATTTTACCGCTGGCTCAACAGCAACAACAGCAAATCCCTCCGTTGCTTCCTCTACAATCATCGTCTATGACTCCCCATAAAACCATGAACGCTTACGCGCCGATGGGATTTTCCACTCCACAGAGCATGATGCACCCTCAAACCCCACAAGCTCTCATGTCTCCTATGGTGAATCGAATGGATCAAAGTCACACGCCCAACGTTCTACCTCCGACGACTCCGGCTCCAATGACACCGATGACGCCTTCGGGCGAACCCAGTTCGATTCCCCAATTGCAAAATATCGTCTCCACTGTCAATTTGGGTTGTAAATTGGATTTGAAAAGGATAGCGCTACACGCGCGAAATGCCGAATACAATCCGAAACGTTTTGCCGCCGTCATAATGCGAATTCGCGAACCACGGACGACCGCGTTGATATTCAGCTCTGGTAAAATGGTTTGTACGGGAGCCAAAAGCGAAGATGATTCTCGTTTGGCTGCTCGAAAATACGCTCGAATTATTCAAAAACTCGGATTTTCCGCGAAATTTTTAGATTTCAAAGTTCAAAATATGGTGGGAAGCTGCGACGTCAAATTTCCCATCCGTTTGGAAGGTTTGGTTCTGACTCACGGTCAATTCAGCAGCTACGAACCCGAACTATTTCCCGGACTCATTTACCGAATGGTCAAACCTAGAATTGTTCTGCTCATATTCGTTTCTGGCAAAGTGGTTTTGACTGGTGCTAAAGTTCGCACAGAAATTTACGAAGCGTTCGACAATATTTACCCTATTTTGAAAAGTTTCAAGAAACAGTGA |
| *UBQ* | 510 | ATGGCTGGATCCGCTCTCAGACGATTAATGGCTGAATATAAGCAGCTCACGTTAAATCCTCCCGAAGGTATTTTAGCGGGTCCCGTCAACGAAGAAAATTTCTTCGAATGGGAAGCGTTAATAACAGGCCCCGAAGGGACGTGTTTCGAAGGTGGTGTATTCCCAACAAAATTGACGTTTCCTCCGGATTATCCGCTGAGCCCTCCTAAAATGCAATTTACCTGTGATATGTTCCATCCTAATATATACGCCGATGGCCGAGTCTGTATTTCCATTCTTCATGCTCCTGGAGATGATCCCATGGGTTATGAGTCAAGCGCTGAACGATGGAGCCCTGTGCAGAGCGTGGAAAAAATTCTCTTATCAGTTGTCAGCATGTTGGCAGAACCAAACGACGAAAGTGGCGCTAATGTCGACGCGGCAAAAATGTGGCGTGAAAACCGCGAAGAATTTAACCAAGTGGCGGAAAGTATCGTTCGTAAAACTTTGGGTATTCCTGACAACGTTTAA |
| **Target gene** | | |
| *OBP3* | 474 | ATGGTTCTCATAGACGGGAAGTCAAGTGTACCTTGGCTCGTAGTGGCTCTCATTGGAGGTTTTCTCGTTGTCCATATAGAATCCAAGGGCTTCACGGAGGAACAGAAAGAAGAATTTGTCAAAGTCATGAAAGAATGTGCAGCGGAGTCGAAGATACCCGAAGCCGAATTTGAAGCGATGACTTCCGAAAGAAAACCTCCAGTGTCGAAAGAAGGAGAGTGTTTCGTCAAATGTATCATGGAAAAAAATGATGTCATTGCGAATAACGAAGTGAATAAAGTGGGCGTAGCTGCGACTCTTGAAGAAATGATTGAAGACAAACCAAAACTAGCGAAGGCCAAAGAAATTCTCGAAGAGTGTACAAAATCAGTCGAGCCACTTGCTAAGGGAGACTCGTGCGAATTTGCTGCGAAATTTGGCGGATGCGTACACTCAAAATTGAAAGATAGCGGTATCATCGGGCCAAAGTTTTAA |

**Supplementary Table 2.** Raw Ct values of twelve candidate genes in different treatments

| **Different treatment** | | **Raw Ct values** | | | | | | | | | | | |
| --- | --- | --- | --- | --- | --- | --- | --- | --- | --- | --- | --- | --- | --- |
|  |  | *actin* | *β-tubulin1* | *β-tubulin2* | *RPL13A* | *EF1α* | *RPS3A* | *GAPDH* | *18sRNA* | *G6PDH* | *EIF4A* | *TBP* | *UBQ* |
| **Developmental stages** | 1 instar | 28.94 | 26.73 | 25.62 | 22.95 | 23.28 | 22.00 | 22.12 | 29.32 | 27.12 | 23.90 | 31.06 | 29.00 |
|  |  | 28.22 | 27.27 | 26.23 | 22.31 | 23.55 | 21.89 | 22.43 | 28.58 | 27.97 | 23.63 | 31.59 | 28.97 |
|  |  | 28.77 | 27.88 | 25.57 | 22.67 | 22.82 | 22.13 | 21.88 | 29.13 | 27.56 | 23.91 | 31.83 | 29.29 |
|  |  | 27.46 | 25.70 | 24.07 | 22.14 | 21.30 | 21.90 | 21.23 | 28.26 | 24.97 | 21.45 | 28.20 | 27.82 |
|  |  | 27.28 | 25.69 | 24.21 | 22.08 | 21.34 | 21.95 | 21.16 | 28.37 | 24.73 | 21.45 | 28.50 | 27.59 |
|  |  | 27.06 | 25.93 | 23.73 | 22.14 | 21.37 | 21.88 | 21.12 | 28.37 | 25.07 | 21.43 | 28.44 | 27.69 |
|  |  | 28.18 | 25.82 | 23.82 | 22.30 | 21.57 | 22.07 | 21.25 | 28.37 | 25.16 | 21.59 | 28.65 | 27.67 |
|  |  | 27.77 | 25.92 | 23.84 | 22.45 | 21.37 | 22.08 | 21.34 | 28.37 | 24.83 | 21.56 | 28.59 | 27.85 |
|  |  | 27.88 | 25.86 | 23.80 | 22.20 | 21.48 | 22.10 | 21.35 | 28.55 | 24.96 | 21.65 | 28.62 | 27.63 |
|  | 2 instar | 28.25 | 25.84 | 23.33 | 23.27 | 22.79 | 23.12 | 23.07 | 29.58 | 28.05 | 23.14 | 29.38 | 28.24 |
|  |  | 28.02 | 26.00 | 23.28 | 23.50 | 22.78 | 23.11 | 22.85 | 29.51 | 27.55 | 23.23 | 29.18 | 28.26 |
|  |  | 28.06 | 25.91 | 23.23 | 23.10 | 22.83 | 23.20 | 22.82 | 29.88 | 27.75 | 23.20 | 29.16 | 28.36 |
|  |  | 27.05 | 25.66 | 23.11 | 23.08 | 22.61 | 23.25 | 22.44 | 29.17 | 27.56 | 23.10 | 29.53 | 28.71 |
|  |  | 27.02 | 25.74 | 23.09 | 22.96 | 22.54 | 23.62 | 22.47 | 28.80 | 27.86 | 23.10 | 29.81 | 28.90 |
|  |  | 27.14 | 25.74 | 23.11 | 23.17 | 22.61 | 23.14 | 22.49 | 28.86 | 27.74 | 23.21 | 29.98 | 28.98 |
|  |  | 27.86 | 25.95 | 22.99 | 23.20 | 23.05 | 23.36 | 22.76 | 28.92 | 27.62 | 23.20 | 29.44 | 28.33 |
|  |  | 27.70 | 25.73 | 22.98 | 23.14 | 22.93 | 23.30 | 22.66 | 29.04 | 27.76 | 23.16 | 29.56 | 28.38 |
|  |  | 27.58 | 25.85 | 22.89 | 23.10 | 23.02 | 23.26 | 22.77 | 29.15 | 27.46 | 23.16 | 29.41 | 28.54 |
|  | 3 instar | 29.29 | 28.25 | 26.27 | 25.50 | 26.08 | 25.17 | 25.36 | 31.71 | 29.63 | 26.19 | 31.95 | 29.91 |
|  |  | 29.57 | 28.88 | 25.70 | 25.39 | 26.17 | 25.23 | 25.32 | 31.81 | 30.09 | 26.16 | 32.16 | 30.08 |
|  |  | 29.94 | 29.08 | 25.83 | 25.42 | 26.11 | 25.58 | 25.24 | 31.63 | 29.49 | 26.14 | 32.18 | 30.18 |
|  |  | 29.14 | 28.65 | 24.98 | 25.11 | 25.91 | 25.38 | 25.02 | 31.11 | 29.92 | 26.31 | 32.53 | 31.09 |
|  |  | 28.95 | 28.03 | 25.15 | 24.96 | 25.75 | 25.22 | 25.06 | 31.01 | 29.96 | 26.39 | 32.74 | 30.36 |
|  |  | 29.15 | 28.03 | 25.05 | 25.02 | 25.75 | 25.24 | 24.98 | 30.90 | 29.52 | 26.12 | 33.21 | 31.53 |
|  |  | 29.99 | 28.45 | 24.66 | 25.25 | 25.62 | 25.40 | 24.76 | 31.69 | 29.48 | 25.92 | 32.01 | 30.16 |
|  |  | 29.47 | 28.22 | 24.69 | 25.32 | 25.54 | 25.37 | 24.64 | 31.10 | 29.67 | 25.84 | 32.34 | 30.28 |
|  |  | 29.90 | 27.77 | 24.64 | 25.21 | 25.60 | 25.40 | 24.95 | 30.78 | 29.55 | 25.79 | 32.34 | 29.96 |
|  | 4 instar | 27.25 | 26.26 | 23.37 | 23.33 | 23.86 | 23.46 | 23.55 | 29.73 | 28.17 | 24.11 | 30.64 | 28.62 |
|  |  | 26.98 | 26.25 | 23.17 | 23.82 | 23.80 | 23.63 | 23.38 | 29.76 | 28.16 | 24.18 | 30.43 | 28.83 |
|  |  | 27.04 | 26.13 | 23.83 | 23.67 | 23.77 | 23.57 | 23.32 | 29.89 | 28.17 | 24.05 | 30.29 | 28.79 |
|  |  | 26.73 | 26.08 | 23.25 | 23.42 | 23.73 | 23.74 | 23.14 | 29.46 | 28.60 | 24.16 | 30.87 | 28.86 |
|  |  | 26.55 | 25.98 | 23.00 | 23.41 | 23.62 | 23.72 | 23.16 | 29.86 | 28.53 | 24.14 | 30.37 | 28.67 |
|  |  | 26.54 | 26.37 | 22.99 | 23.49 | 23.64 | 23.50 | 23.13 | 29.54 | 28.10 | 24.22 | 30.97 | 29.07 |
|  |  | 27.14 | 26.19 | 23.05 | 23.49 | 24.13 | 23.83 | 23.57 | 29.59 | 28.53 | 24.54 | 30.58 | 28.67 |
|  |  | 27.29 | 26.36 | 23.10 | 23.52 | 24.23 | 23.88 | 23.52 | 29.54 | 28.86 | 24.57 | 30.43 | 28.49 |
|  |  | 27.29 | 26.54 | 22.93 | 23.49 | 24.25 | 24.02 | 23.51 | 29.57 | 28.67 | 24.53 | 30.71 | 28.32 |
|  | 5 instar | 28.18 | 26.82 | 24.15 | 24.14 | 24.39 | 24.20 | 23.87 | 29.87 | 28.95 | 24.15 | 30.49 | 29.42 |
|  |  | 27.91 | 26.75 | 24.13 | 24.16 | 24.33 | 24.10 | 23.76 | 29.92 | 28.37 | 24.14 | 30.68 | 29.16 |
|  |  | 27.96 | 26.71 | 24.17 | 24.10 | 24.25 | 24.24 | 23.88 | 30.10 | 28.45 | 24.15 | 30.67 | 29.18 |
|  |  | 28.24 | 27.14 | 24.24 | 24.16 | 24.28 | 24.34 | 23.76 | 29.62 | 29.15 | 24.52 | 30.92 | 29.42 |
|  |  | 28.01 | 27.10 | 24.43 | 24.29 | 24.31 | 24.33 | 23.65 | 29.78 | 29.11 | 24.38 | 30.17 | 29.17 |
|  |  | 28.03 | 27.19 | 24.28 | 24.30 | 24.25 | 24.20 | 23.54 | 30.14 | 29.45 | 24.45 | 30.95 | 29.31 |
|  |  | 28.88 | 27.19 | 24.10 | 24.08 | 25.27 | 24.68 | 25.19 | 30.61 | 30.03 | 25.45 | 31.15 | 29.25 |
|  |  | 28.61 | 27.13 | 24.12 | 24.10 | 25.40 | 24.73 | 25.00 | 30.38 | 29.93 | 25.48 | 31.55 | 29.33 |
|  |  | 28.92 | 27.20 | 24.01 | 23.99 | 25.53 | 25.07 | 25.12 | 30.81 | 29.82 | 25.41 | 31.91 | 29.60 |
|  | adult | 29.71 | 27.63 | 26.00 | 24.28 | 24.89 | 25.01 | 24.00 | 29.22 | 28.57 | 24.66 | 28.96 | 28.19 |
|  |  | 29.50 | 27.52 | 25.97 | 24.49 | 24.72 | 25.00 | 23.66 | 29.50 | 28.72 | 24.82 | 28.70 | 27.94 |
|  |  | 29.52 | 27.49 | 26.00 | 24.38 | 24.61 | 25.03 | 23.71 | 29.16 | 28.22 | 24.60 | 28.99 | 28.00 |
|  |  | 29.11 | 27.52 | 25.73 | 24.39 | 24.44 | 25.16 | 23.39 | 28.48 | 28.29 | 24.68 | 29.24 | 28.99 |
|  |  | 29.00 | 27.50 | 25.72 | 24.42 | 24.47 | 25.21 | 23.37 | 28.45 | 28.03 | 24.91 | 29.37 | 28.86 |
|  |  | 29.19 | 27.55 | 25.42 | 24.21 | 24.43 | 25.01 | 23.39 | 28.77 | 28.14 | 24.80 | 29.94 | 28.69 |
|  |  | 30.00 | 27.73 | 25.68 | 24.44 | 25.38 | 25.31 | 24.23 | 28.84 | 28.72 | 25.61 | 29.87 | 28.27 |
|  |  | 29.82 | 27.80 | 25.83 | 24.40 | 25.54 | 25.21 | 24.19 | 28.83 | 29.06 | 25.53 | 29.75 | 28.22 |
|  |  | 29.66 | 27.72 | 25.60 | 24.41 | 25.39 | 25.26 | 24.25 | 28.67 | 28.91 | 25.37 | 29.96 | 28.22 |
| **Tissues** | head | 27.02 | 24.08 | 23.37 | 22.31 | 21.66 | 22.29 | 21.17 | 29.21 | 26.21 | 22.17 | 28.14 | 26.24 |
|  |  | 26.69 | 24.76 | 23.30 | 22.31 | 21.59 | 22.17 | 20.90 | 29.30 | 26.10 | 22.26 | 28.19 | 27.08 |
|  |  | 27.14 | 24.25 | 23.26 | 22.21 | 21.70 | 22.56 | 20.86 | 29.22 | 26.19 | 22.14 | 28.24 | 26.33 |
|  |  | 26.21 | 24.09 | 23.15 | 22.05 | 21.52 | 22.89 | 20.91 | 29.03 | 26.38 | 22.53 | 28.75 | 26.71 |
|  |  | 26.11 | 23.96 | 23.31 | 22.12 | 21.58 | 22.75 | 20.73 | 29.06 | 26.25 | 22.28 | 28.68 | 26.51 |
|  |  | 26.56 | 24.08 | 22.90 | 22.12 | 21.58 | 22.54 | 21.00 | 29.01 | 26.39 | 22.39 | 28.55 | 27.17 |
|  |  | 27.32 | 24.23 | 23.16 | 22.17 | 23.14 | 23.42 | 22.00 | 30.22 | 27.70 | 23.52 | 30.05 | 27.31 |
|  |  | 27.06 | 24.33 | 23.21 | 22.15 | 23.21 | 23.05 | 21.83 | 30.02 | 27.94 | 23.54 | 30.19 | 27.34 |
|  |  | 27.47 | 24.20 | 23.21 | 22.24 | 23.20 | 23.46 | 22.16 | 30.23 | 27.61 | 23.44 | 30.20 | 27.70 |
|  | thorax | 28.84 | 27.12 | 26.03 | 24.94 | 25.31 | 25.19 | 23.62 | 31.29 | 26.77 | 25.30 | 32.23 | 29.15 |
|  |  | 28.60 | 27.19 | 25.96 | 25.00 | 25.46 | 25.18 | 23.43 | 31.24 | 26.70 | 25.23 | 31.67 | 29.10 |
|  |  | 28.85 | 27.22 | 26.03 | 24.96 | 25.44 | 25.42 | 23.51 | 31.21 | 26.69 | 25.27 | 32.33 | 29.24 |
|  |  | 28.80 | 27.44 | 26.17 | 25.07 | 25.44 | 25.65 | 23.39 | 31.30 | 27.15 | 25.95 | 32.71 | 29.50 |
|  |  | 28.63 | 27.55 | 26.10 | 25.17 | 25.33 | 25.41 | 23.44 | 31.00 | 27.17 | 25.55 | 32.21 | 29.52 |
|  |  | 29.67 | 27.66 | 25.97 | 25.17 | 25.48 | 25.58 | 24.48 | 31.20 | 27.09 | 25.62 | 32.55 | 29.98 |
|  |  | 29.62 | 27.83 | 26.27 | 25.03 | 25.15 | 25.65 | 23.33 | 31.14 | 27.33 | 26.12 | 31.51 | 29.31 |
|  |  | 29.28 | 27.69 | 26.19 | 24.97 | 25.17 | 25.56 | 23.32 | 31.00 | 27.13 | 25.36 | 31.68 | 29.15 |
|  |  | 30.29 | 28.27 | 26.06 | 25.12 | 26.23 | 26.06 | 24.23 | 30.96 | 27.14 | 25.34 | 31.27 | 29.21 |
|  | abdomen | 27.87 | 28.02 | 25.47 | 23.46 | 23.83 | 24.25 | 23.02 | 28.67 | 26.09 | 23.74 | 29.19 | 27.83 |
|  |  | 27.75 | 28.13 | 25.53 | 23.50 | 24.13 | 24.10 | 22.71 | 28.66 | 26.03 | 23.80 | 29.15 | 27.49 |
|  |  | 27.90 | 28.05 | 25.37 | 23.45 | 24.05 | 24.17 | 22.85 | 28.38 | 26.03 | 24.16 | 29.26 | 27.44 |
|  |  | 27.97 | 28.74 | 26.15 | 23.89 | 24.16 | 24.62 | 23.06 | 28.85 | 26.73 | 24.35 | 29.23 | 27.45 |
|  |  | 28.34 | 29.27 | 26.19 | 23.86 | 24.16 | 24.67 | 22.79 | 29.11 | 26.30 | 24.14 | 29.41 | 27.66 |
|  |  | 27.70 | 28.86 | 26.04 | 24.10 | 24.24 | 25.05 | 22.86 | 29.22 | 26.40 | 24.06 | 29.39 | 27.70 |
|  |  | 27.90 | 28.37 | 25.83 | 23.55 | 24.14 | 24.19 | 22.74 | 28.75 | 27.27 | 24.33 | 29.30 | 27.46 |
|  |  | 27.41 | 28.39 | 25.81 | 23.98 | 24.21 | 24.21 | 22.84 | 28.72 | 26.69 | 24.15 | 29.06 | 27.52 |
|  |  | 27.36 | 28.60 | 26.14 | 23.81 | 24.17 | 24.21 | 23.07 | 29.04 | 26.55 | 24.06 | 28.69 | 27.50 |
|  | antenna | 26.77 | 24.95 | 22.25 | 22.22 | 21.74 | 22.23 | 20.70 | 27.45 | 25.00 | 22.26 | 28.11 | 25.58 |
|  |  | 26.42 | 24.74 | 22.16 | 22.24 | 21.87 | 22.05 | 20.76 | 27.14 | 25.12 | 22.28 | 28.27 | 25.70 |
|  |  | 26.50 | 25.05 | 22.15 | 22.43 | 21.64 | 21.97 | 20.57 | 27.13 | 25.10 | 22.21 | 27.87 | 25.92 |
|  |  | 26.11 | 24.60 | 22.30 | 22.15 | 21.40 | 22.13 | 21.00 | 27.46 | 24.77 | 22.14 | 27.72 | 25.43 |
|  |  | 26.36 | 25.16 | 22.51 | 22.27 | 21.64 | 22.12 | 20.88 | 27.17 | 24.63 | 22.08 | 27.98 | 25.46 |
|  |  | 26.18 | 24.60 | 22.37 | 22.20 | 21.69 | 22.13 | 20.80 | 27.36 | 24.58 | 22.27 | 28.13 | 25.51 |
|  |  | 26.30 | 25.41 | 21.76 | 22.67 | 21.21 | 22.58 | 20.90 | 27.42 | 25.37 | 22.21 | 27.66 | 26.17 |
|  |  | 26.27 | 25.11 | 21.49 | 22.54 | 21.33 | 22.94 | 20.96 | 27.51 | 25.09 | 22.29 | 28.04 | 26.44 |
|  |  | 26.32 | 25.48 | 21.78 | 22.45 | 21.40 | 22.78 | 20.89 | 27.73 | 25.07 | 22.26 | 27.72 | 26.16 |
|  | leg | 28.80 | 29.46 | 25.23 | 24.02 | 23.99 | 24.96 | 22.91 | 31.31 | 28.38 | 23.49 | 30.52 | 26.35 |
|  |  | 28.61 | 28.92 | 25.01 | 24.00 | 24.67 | 24.56 | 23.03 | 30.82 | 28.74 | 22.43 | 30.32 | 28.94 |
|  |  | 28.68 | 29.16 | 25.04 | 24.25 | 24.47 | 24.56 | 22.79 | 30.40 | 28.26 | 23.04 | 29.99 | 27.77 |
|  |  | 27.48 | 28.38 | 24.58 | 23.74 | 23.42 | 24.18 | 22.31 | 30.10 | 27.17 | 22.97 | 30.52 | 27.94 |
|  |  | 27.38 | 28.31 | 24.54 | 23.70 | 23.24 | 24.01 | 22.17 | 29.95 | 27.14 | 23.06 | 30.40 | 28.03 |
|  |  | 27.47 | 28.45 | 24.46 | 23.72 | 23.32 | 24.07 | 22.26 | 30.15 | 27.04 | 22.92 | 31.03 | 28.06 |
|  |  | 27.09 | 28.01 | 23.96 | 23.31 | 23.25 | 23.54 | 22.35 | 29.80 | 27.40 | 22.89 | 30.61 | 27.71 |
|  |  | 27.19 | 28.26 | 23.99 | 23.22 | 23.27 | 23.87 | 22.35 | 29.71 | 27.18 | 23.11 | 30.53 | 27.88 |
|  |  | 27.47 | 28.60 | 24.35 | 23.42 | 23.41 | 23.68 | 22.45 | 29.71 | 27.26 | 22.88 | 30.63 | 27.76 |
|  | wing | 27.49 | 27.37 | 23.03 | 23.27 | 22.86 | 23.30 | 20.21 | 28.67 | 25.61 | 22.63 | 28.90 | 26.35 |
|  |  | 27.26 | 27.12 | 23.09 | 23.18 | 22.72 | 23.50 | 20.60 | 28.49 | 25.17 | 22.95 | 27.91 | 27.01 |
|  |  | 27.18 | 26.93 | 23.06 | 23.12 | 22.93 | 23.41 | 20.32 | 28.53 | 25.03 | 22.78 | 27.72 | 26.24 |
|  |  | 27.14 | 27.46 | 23.21 | 23.13 | 22.59 | 23.38 | 20.51 | 28.58 | 25.29 | 22.69 | 28.37 | 26.81 |
|  |  | 26.46 | 26.86 | 23.13 | 23.09 | 22.47 | 23.16 | 20.13 | 28.35 | 25.12 | 22.69 | 28.49 | 27.17 |
|  |  | 26.77 | 27.16 | 23.31 | 23.35 | 22.53 | 23.35 | 20.28 | 28.82 | 25.19 | 22.87 | 28.55 | 26.56 |
|  |  | 27.41 | 28.26 | 23.89 | 24.53 | 24.04 | 24.77 | 21.35 | 29.39 | 26.78 | 24.16 | 29.57 | 27.77 |
|  |  | 27.62 | 28.17 | 23.86 | 24.48 | 23.78 | 24.73 | 21.30 | 29.44 | 26.63 | 24.03 | 29.74 | 28.02 |
|  |  | 27.56 | 28.33 | 23.97 | 24.53 | 23.93 | 24.76 | 21.41 | 29.72 | 26.74 | 24.12 | 29.98 | 28.00 |
| **Sexes** | adultF | 27.67 | 24.50 | 21.05 | 19.78 | 18.37 | 19.57 | 18.11 | 24.23 | 21.15 | 19.88 | 23.60 | 23.57 |
|  |  | 27.50 | 24.45 | 20.99 | 19.69 | 18.34 | 19.83 | 17.93 | 24.42 | 21.10 | 19.77 | 23.61 | 23.74 |
|  |  | 27.58 | 24.70 | 21.06 | 19.68 | 18.48 | 19.78 | 18.02 | 24.26 | 21.22 | 19.84 | 24.07 | 23.60 |
|  |  | 27.85 | 24.33 | 20.97 | 19.51 | 18.26 | 19.44 | 17.95 | 24.07 | 21.17 | 19.80 | 23.91 | 23.59 |
|  |  | 27.57 | 24.41 | 20.92 | 19.40 | 18.26 | 19.34 | 17.86 | 24.28 | 21.19 | 19.48 | 24.00 | 23.32 |
|  |  | 27.53 | 24.28 | 20.84 | 19.42 | 18.18 | 19.51 | 17.97 | 24.14 | 21.16 | 19.69 | 23.73 | 23.09 |
|  |  | 27.75 | 24.10 | 20.81 | 19.46 | 18.20 | 19.32 | 17.94 | 24.04 | 20.86 | 19.48 | 23.51 | 23.29 |
|  |  | 27.51 | 23.91 | 20.80 | 19.36 | 18.13 | 19.32 | 17.73 | 23.89 | 20.90 | 19.48 | 23.39 | 23.05 |
|  |  | 27.46 | 24.08 | 20.62 | 19.33 | 18.21 | 19.42 | 17.78 | 24.08 | 20.93 | 19.44 | 23.64 | 23.16 |
|  | adultM | 30.05 | 26.94 | 21.70 | 21.93 | 20.42 | 22.01 | 18.96 | 27.41 | 22.47 | 21.90 | 27.03 | 25.22 |
|  |  | 29.72 | 26.97 | 21.67 | 21.88 | 20.38 | 22.01 | 18.78 | 27.34 | 22.48 | 21.83 | 27.10 | 25.25 |
|  |  | 29.57 | 26.84 | 21.60 | 21.89 | 20.42 | 22.06 | 18.77 | 27.34 | 22.48 | 21.84 | 27.14 | 25.30 |
|  |  | 30.04 | 26.77 | 21.55 | 21.93 | 20.31 | 21.77 | 18.70 | 27.22 | 22.24 | 21.70 | 27.04 | 24.57 |
|  |  | 29.63 | 26.67 | 21.54 | 21.78 | 20.30 | 21.77 | 18.66 | 27.28 | 22.22 | 21.49 | 27.22 | 24.93 |
|  |  | 29.50 | 26.65 | 21.60 | 21.83 | 20.18 | 21.93 | 18.69 | 27.23 | 22.26 | 21.65 | 27.04 | 24.80 |
|  |  | 30.16 | 26.33 | 21.74 | 21.83 | 20.28 | 21.87 | 18.66 | 27.25 | 22.16 | 21.69 | 27.20 | 25.04 |
|  |  | 29.84 | 26.42 | 21.44 | 21.82 | 20.20 | 21.75 | 18.63 | 27.16 | 22.16 | 21.99 | 26.83 | 25.02 |
|  |  | 29.83 | 26.38 | 21.41 | 21.74 | 20.28 | 21.86 | 18.58 | 27.30 | 22.27 | 21.79 | 27.06 | 25.10 |
|  | thoraxF | 31.92 | 26.94 | 26.57 | 22.14 | 21.22 | 21.89 | 20.32 | 27.00 | 25.22 | 22.74 | 34.03 | 26.56 |
|  |  | 31.63 | 26.41 | 26.83 | 22.05 | 21.17 | 21.96 | 20.01 | 26.92 | 24.87 | 22.54 | 33.74 | 26.37 |
|  |  | 31.72 | 26.81 | 26.51 | 21.94 | 21.23 | 22.27 | 19.88 | 26.93 | 25.07 | 22.39 | 33.96 | 26.54 |
|  |  | 31.96 | 26.57 | 28.06 | 23.06 | 22.43 | 22.76 | 21.45 | 27.94 | 26.17 | 23.25 | 31.08 | 27.09 |
|  |  | 31.51 | 26.43 | 27.60 | 22.83 | 22.26 | 22.75 | 21.21 | 27.96 | 26.19 | 23.22 | 30.85 | 27.05 |
|  |  | 31.45 | 26.82 | 27.33 | 22.76 | 22.19 | 22.78 | 21.17 | 27.83 | 26.20 | 23.11 | 30.65 | 27.05 |
|  |  | 32.06 | 26.41 | 27.26 | 22.66 | 21.58 | 22.40 | 20.37 | 26.93 | 24.02 | 22.46 | 27.36 | 25.46 |
|  |  | 31.54 | 26.22 | 27.04 | 22.47 | 21.49 | 22.43 | 20.16 | 26.73 | 24.17 | 22.47 | 27.16 | 25.82 |
|  |  | 31.37 | 26.18 | 27.39 | 22.33 | 21.49 | 22.44 | 20.12 | 26.62 | 24.03 | 22.41 | 27.22 | 25.96 |
|  | thoraxM | 32.93 | 26.03 | 29.95 | 22.63 | 20.96 | 22.41 | 20.05 | 27.87 | 22.53 | 22.24 | 27.28 | 25.18 |
|  |  | 32.51 | 25.92 | 30.15 | 22.47 | 20.80 | 22.52 | 19.74 | 28.00 | 22.62 | 21.95 | 27.42 | 25.17 |
|  |  | 32.51 | 25.59 | 28.91 | 22.53 | 21.03 | 22.43 | 19.63 | 27.99 | 22.65 | 21.84 | 27.27 | 25.19 |
|  |  | 32.73 | 28.13 | 30.44 | 23.23 | 22.59 | 23.09 | 22.21 | 28.71 | 24.29 | 23.16 | 28.58 | 26.01 |
|  |  | 32.24 | 28.24 | 30.03 | 23.02 | 22.62 | 23.06 | 22.08 | 28.59 | 24.24 | 23.10 | 28.97 | 25.67 |
|  |  | 32.26 | 28.21 | 30.35 | 23.06 | 22.70 | 22.98 | 22.10 | 28.55 | 24.30 | 22.87 | 28.75 | 25.81 |
|  |  | 31.50 | 26.44 | 28.88 | 22.64 | 21.19 | 22.45 | 19.90 | 27.79 | 22.76 | 22.18 | 27.94 | 25.51 |
|  |  | 31.50 | 26.30 | 28.89 | 22.65 | 21.18 | 22.56 | 19.74 | 27.95 | 22.85 | 22.12 | 28.30 | 25.59 |
|  |  | 31.45 | 26.13 | 29.21 | 22.46 | 21.24 | 22.52 | 19.71 | 27.80 | 22.87 | 22.12 | 27.76 | 25.56 |

Note: Each sample data is 3 biological replications × 3 technical duplications, a total of 9 data.
